# Optic nerve regeneration requires the intracellular domain of LIFR**α**/CD118

**DOI:** 10.1172/jci.insight.202223

**Published:** 2026-09-01

**Authors:** Qian Jiang, Cong Wang, Yuerong Ren, Peiyun Duan, Ke Tian, Xiangwei Duan, Binghan Cai, Changzhong Xu, Ke Liu, Jian Li, Larry Benowitz, Ningli Wang, Bing Jiang, Lili Xie

**Affiliations:** 1 Department of Ophthalmology, The Second Xiangya Hospital, Central South University, Changsha, Hunan, China.; 2 Hunan Clinical Research Center of Ophthalmic Disease, Changsha, Hunan, China.; 3 Division of Rheumatology, Inflammation and Immunity, Brigham and Women’s Hospital and Department of Medicine, Harvard Medical School, Boston, Massachusetts, USA.; 4 Departments of Neurosurgery and Ophthalmology, Boston Children’s Hospital and Harvard Medical School, Boston, Massachusetts, USA.; 5 Department of Ophthalmology, Beijing Tongren Hospital, Capital Medical University, Beijing, China.

**Keywords:** Neuroscience, Ophthalmology, Gene therapy, Growth factors, Neurodegeneration

## Abstract

Identifying factors that govern the ability of retinal ganglion cells (RGCs) to extend axons is an important step in developing therapies to achieve recovery after optic nerve injury. Here, we report that the intracellular domain of the leukemia inhibitory factor receptor (LIFR/CD118) is essential for mature RGCs’ ability to regenerate injured axons independent of the cognate ligand (LIF) and other therapies. Overexpression of LIFR in adult RGCs induces neurite outgrowth in cultured RGCs and axon regeneration in vivo while strongly amplifying RGCs’ response to LIF itself and to unrelated growth factors. Conversely, downregulation of LIFR strongly suppresses the pro-regenerative effects of PTEN knockdown and other potent stimuli. LIFR modulation alters the constitutive activity of the MAPK pathway, in contrast to LIF itself, which primarily activates pSTAT3. The extracellular domain–truncated LIFR construct retains substantial pro-regenerative activity, whereas mutation of intracellular signaling motifs reduces the full regenerative effect of LIFR. Together, these findings identify LIFR as a key cell-autonomous regulator of optic nerve regeneration in mature RGCs.

## Abstract 2

LIFR promotes optic nerve regeneration through its intracellular domain and enhances the ability of RGCs to respond to other regenerative treatments.

## Graphical abstract

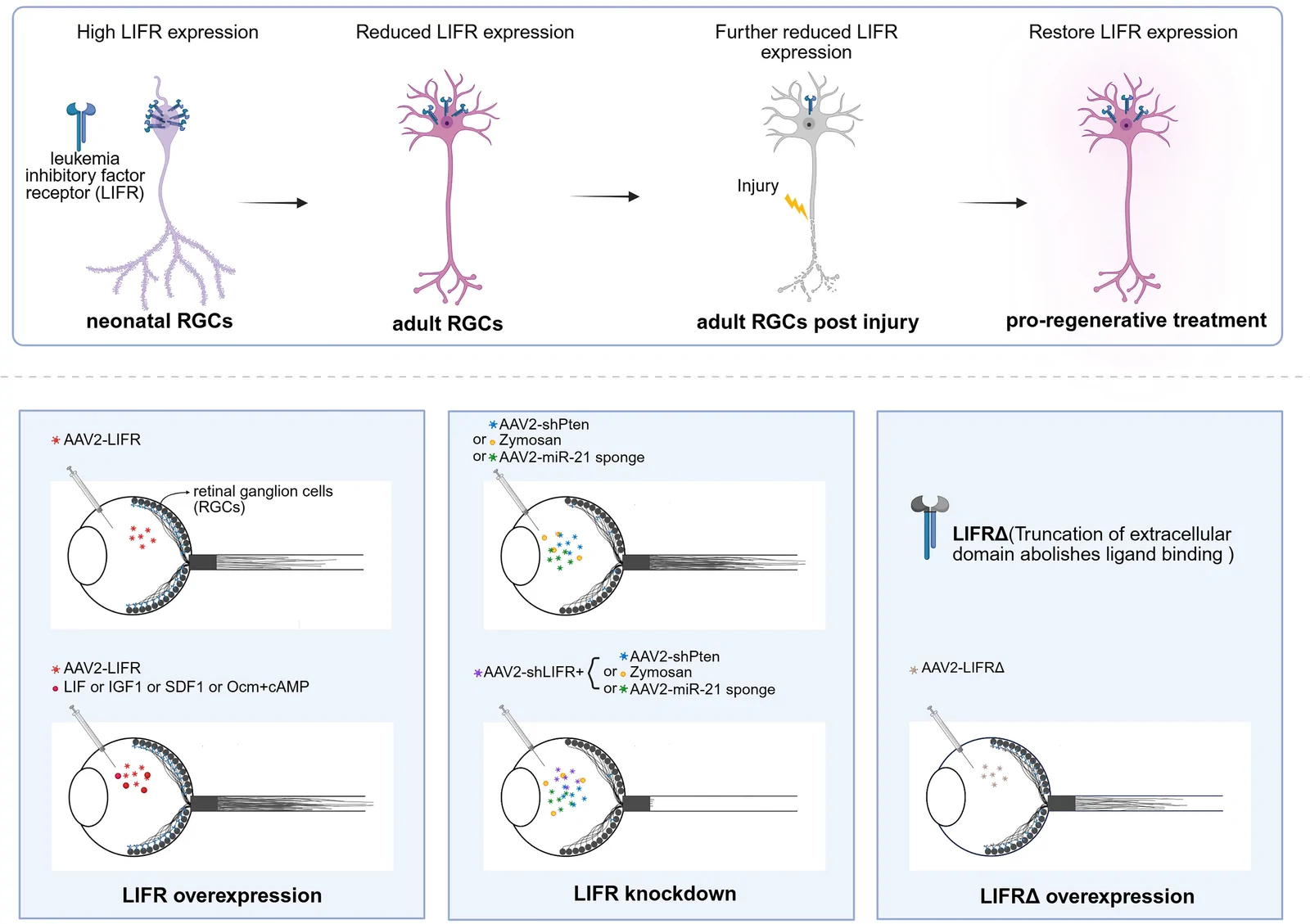

## Introduction

In mammals, the regenerative ability and trophic responsiveness of retinal ganglion cells (RGCs) decline after birth (1–3), contributing to the irreversible visual losses commonly seen after traumatic or ischemic optic nerve injury and in several neuro-ophthalmic conditions, including glaucoma. Along with other important changes known to occur over the course of RGC development (4–6), the downregulation of critical receptors for trophic factors could potentially play a role in limiting recovery. Because several previously unrecognized trophic factors and their cognate receptors have been shown to stimulate regeneration of the injured optic nerve and to synergize with other treatments (6–14), we have extended the search for other important ligands and receptors. Receptors are highly specialized biological transducers that receive signals and respond to outside stimuli, commonly the cognate ligands. By screening the multiple receptors found to be expressed in adult RGCs in our earlier RNA-seq analysis (6), we discovered that expression of the leukemia inhibitory factor receptor (LIFR) correlates with the decline of RGCs’ regenerative potential. LIFR belongs to the type I cytokine receptor family, acting as a receptor for LIF and the related proteins oncostatin M (OSM) and cardiotrophin 1 (CTF1) (15). Prior studies have documented divergent regenerative outcomes in response to LIF administration alone (7, 16). By contrast, our work identifies its cognate receptor, LIFR, as a critical, ligand-independent modulator of optic nerve regeneration that is indispensable for the therapeutic efficacy of unrelated pro-regenerative treatments. Consistent with these observations, LIFR overexpression and LIFR knockdown elicit opposing transcriptional alterations in mature RGCs.

## Results

### LIFR expression correlates with RGC growth potential

#### LIFR expression after optic nerve injury and during regeneration.

To investigate whether the expression of any receptors correlates with RGCs’ growth state, we first examined receptor expression profiles in RGCs after injury and under regenerative conditions. RGCs were FACS-sorted from mature mice at 1, 3, or 5 days after optic nerve crush (ONC) with or without intraocular injection of zymosan, a yeast cell wall preparation that induces optic nerve regeneration by elevating intraocular inflammation (Supplemental Figure 1D; supplemental material available online with this article; https://doi.org/10.1172/jci.insight.202223DS1) and levels of immune cell–derived growth factors (e.g., oncomodulin, SDF1) (8–10, 17, 18). These studies used *Vglut2*-tdTomato mice in which RGCs were genetically labeled, as verified by immunostaining against the RGC-selective marker βIII-tubulin (Supplemental Figure 1A). Using qPCR to assess expression profiles of 53 receptors previously found by RNA-seq to be expressed in normal adult RGCs (6), only the LIF receptor (*Lifr*) showed a significant decrease after ONC and partial recovery with the pro-regenerative treatment (Table 1). In a follow-up study, we found that *Lifr* mRNA in mature RGCs was about half that seen at P5 (*P* < 0.01; Supplemental Figure 1B) and, in conformity with the results reported above, was further reduced by 57% from normal adult levels at day 3 after ONC (*P* < 0.01) and by 97% at day 5 after ONC (*P* < 0.0001) (Supplemental Figure 1C). ONC-induced reduction of *Lifr* mRNA was partially alleviated by intraocular zymosan (compared with ONC-only groups, intravitreal zymosan enhanced *Lifr* mRNA levels in adult RGCs 4.0-fold at day 3, *P* < 0.0001; and 3.6-fold at day 5, *P* < 0.001) (Supplemental Figure 1D). At the protein level, compared with RGCs from mice at P1, LIFR intensity declined by approximately 30% 7 days after birth (*P* < 0.0001; Figure 1, A and B) but did not decrease further at later time points (all *P* > 0.05; Figure 1, A and B, and Supplemental Figure 1, E and F; RGCs immunostained for RNA-binding protein with multiple splicing, RBPMS). In parallel with the decline in *Lifr* mRNA levels, optic nerve injury further reduced LIFR intensity in mature RGCs by 25%–32% (25.3% reduction at day 1 after ONC, *P* < 0.05; 31.6% reduction at day 5 after ONC, *P* < 0.01) (Figure 1, A and B). The consistent disparity between the mRNA and protein changes suggests that the LIFR protein may turn over slowly.

To characterize developmental changes in LIFR expression in greater detail, we assessed LIFR protein levels in rats at E17, E21, P1, P2, P8, and P11, time points previously used to study developmental changes in RGCs’ growth potential (19). In conformity with the finding that embryonic RGCs have a stronger intrinsic capacity for axon growth than postnatal RGCs (19), we saw a 16.5% decrease in LIFR expression between E17 and E21 that remained at a similar level through P2 (at P1 and P2, *P* < 0.05), then declined further by P8 (55.9% decline relative to E17 at P8, 52.2% decline at P11, both *P* < 0.0001; Supplemental Figure 1, G and H).

#### LIFR overexpression is sufficient to stimulate neurite outgrowth in adult RGCs.

To evaluate the efficacy of LIFR overexpression, we delivered CAG promoter-driven adeno-associated virus serotype 2.2 (AAV2-LIFR) via intravitreal injection. This viral vector preferentially targets RGCs, with minor off-target transduction of amacrine cells and glia (11, 20, 21). Retinal flat-mount analysis showed that intravitreal AAV2 transduced approximately 82.7% of RBPMS^+^ RGCs (Supplemental Figure 2A). Within RBPMS^+^ RGCs, AAV2-LIFR induced a 3.2-fold elevation in LIFR protein abundance compared with the empty vector controls (*P* < 0.01; Figure 1C and Supplemental Figure 2B). Western blot analysis of whole retinas obtained 2 weeks after viral injection failed to detect a significant rise in total LIFR protein (*P* > 0.05; Supplemental Figure 2, C and D), presumably because RGCs represent less than 1% of all cells in the retina, but at the mRNA level, whole-retina qPCR showed a modest yet significant 13.5% upregulation of endogenous *Lifr* transcripts after AAV2-LIFR delivery, consistent with our quantitative immunofluorescence measurements (*P* < 0.05; Supplemental Figure 2E).

Next, to test the consequences of LIFR overexpression, we again overexpressed LIFR (versus a control construct) in adult *Vglut2*-tdTomato mice (in which RGCs are genetically labeled with tdTomato), and then prepared dissociated retinal cultures 2 weeks later. After 3 days in culture, mature RGCs overexpressing LIFR showed a 2.4-fold increase in neurite outgrowth compared with controls (*P* < 0.01; Figure 1, D and E). Regarding the cognate ligand, in the absence of receptor overexpression, LIF at either 100 or 300 ng/mL had no detectable effect on neurite outgrowth nor RGC survival (*P* > 0.05; Figure 1, D and E, and Supplemental Figure 2F). However, with LIFR overexpression, LIF approximately doubled levels of neurite outgrowth: 120% increase with LIF at 100 ng/mL (*P* < 0.0001) and 140% increase with LIF at 300 ng/mL (*P* < 0.01; Figure 1, D and E). In one case, we detected a 24.3% decrease in RGC survival in the presence of the ligand (AAV2-LIFR + LIF, 100 ng/mL, *P* < 0.05; Supplemental Figure 2F).

#### Intraocular zymosan stimulation robustly upregulates Lif mRNA levels in infiltrating neutrophils.

Because neutrophils recruited by intraocular zymosan secrete trophic factors that promote optic nerve regeneration (9, 10, 17), we tested whether these cells also express *Lif* transcripts. By immunochemistry for the neutrophil marker Gr1, we confirmed the presence of numerous neutrophils in the vitreous chamber from 12 hours to 3 days after intraocular zymosan, which declined by 5 days (Supplemental Figure 3A). We isolated neutrophils from the vitreous using magnetic beads coated with an anti-Ly6G antibody at different time points after intraocular zymosan and assessed neutrophil purity by flow cytometry after incubation with an anti-Gr1 primary antibody conjugated with FITC. Neutrophils, characterized by Giemsa-stained multilobed nuclei, accounted for 93.8% of the isolated cells (Supplemental Figure 3B). Because the number of isolated neutrophils was limited, we quantified *Lif* mRNA by absolute qPCR. Compared with the sham-treated group, *Lif* mRNA levels increased dramatically within the first 5 days after zymosan injection (50.4-fold increase at day 1, *P* > 0.05; 214.8-fold increase at day 3, *P* < 0.0001; 52.6-fold increase at day 5, *P* > 0.05; Supplemental Figure 3C). Thus, infiltrative neutrophils express *Lif* mRNA in addition to the trophic factors reported previously (7, 9, 14, 18).

### LIFR overexpression induces optic nerve regeneration with minimal reliance on its canonical ligands

#### AAV2-LIFR promotes axon regeneration.

To investigate the effect of LIFR overexpression in vivo, we injected AAV2-LIFR intraocularly into adult mice 2 weeks prior to ONC. Even in the absence of exogenous LIF, LIFR overexpression augmented axon regeneration 4.5-fold over mice receiving a control vector when evaluated 2 weeks after nerve injury (*P* < 0.0001; Figure 2, A and B) without altering RGC survival (*P* > 0.05; Figure 2, A and C). As previously reported (6, 8, 9, 22), RGC survival in whole-mounted retina was visualized by immunostaining against βIII-tubulin (antibody TUJ1). We verified that TUJ1 immunoreactivity was restricted to RBPMS^+^ RGCs (Supplemental Figure 4A).

#### AAV2-LIFR–induced regeneration is sustained after neutralization of the LIFR ligand.

To determine whether the effect of LIFR overexpression requires the presence of LIF, the cognate ligand, we introduced a LIF-neutralizing antibody to diminish endogenous levels of the protein. LIF concentration in aqueous humor and serum is approximately 4 pg/mL (0.15 pM: human) (23), and we used a more than 10,000-fold excess of LIF neutralizing antibody (400 ng/mL, ~2.7 nM) to reduce levels of the protein. As before, AAV2-LIFR was introduced intraocularly 2 weeks before ONC, and the LIF neutralizing antibody (400 ng/mL) was injected intravitreally twice, once at 5 days before and again immediately after ONC. LIF neutralizing antibody did not alter AAV2-LIFR–induced axon regeneration (*P* > 0.05; Figure 2, A and B) and had no impact on RGC survival (*P* > 0.05; Figure 2, A and C). We also tested the effect of applying neutralizing antibodies against 2 alternative ligands for LIFR, cardiotrophin-1 (anti-CTF1; 400 ng/mL) and oncostatin M (anti-OSM; 3000 ng/mL) in the vitreous. Neither antibody altered AAV2-LIFR–induced axon regeneration (*P* > 0.05; Supplemental Figure 4, B–D).

#### LIFR overexpression augments the effects of LIF.

In complementary studies, we investigated whether LIFR overexpression improves RGCs’ response to the cognate ligand. Whereas a single intraocular injection of LIF (1 μg/eye) immediately after ONC had no detectable effect (*P* > 0.05 compared with BSA, 1 μg/eye; Figure 2, D and E), it had a clear effect when LIFR was overexpressed (intraocular AAV2-LIFR: 3.2-fold increase compared with LIF plus AAV2-Ctrl, Figure 2, D and E; 2.4-fold increase compared with AAV2-LIFR: both *P* < 0.0001, Figure 2, A, B, D, and E). RGC survival was unaffected in all cases (*P* > 0.05; Figure 2, A, C, D, and F).

### AAV2-LIFR enhances RGCs’ response to unrelated growth factors

We next examined whether receptor overexpression also increases RGCs’ responsiveness to unrelated trophic factors, including IGF1 (1 μg/eye), stromal cell–derived factor 1 (SDF1, 0.9 μg/eye), and oncomodulin (Ocm, 90 ng/eye), all of which have been shown to promote optic nerve regeneration (7, 9, 11, 14). As reported earlier (7, 9, 14), single intraocular injections of either IGF1 or SDF1 stimulated moderate levels of axon regeneration (IGF1: *P* < 0.01, Figure 3, A and B; SDF1: *P* < 0.0001, Figure 3, A and D), and strongly increased RGC survival (72.4% increase for IGF1, *P* < 0.001, Figure 3C; 81.3% increase for SDF1, *P* < 0.0001, Figure 3E), whereas Ocm (plus CPT-cAMP) had little effect in the absence of complementary factors (e.g., *Pten* deletion, SDF1 expression) (7) (*P* > 0.05, Figure 3F). However, when combined with LIFR overexpression, all 3 factors roughly doubled levels of axon regeneration compared with receptor overexpression alone (*P* < 0.05; Figure 3, A, B, D, and F). Thus, LIFR elevation enhances RGCs’ responsiveness to both the cognate ligand and to unrelated trophic factors. None of the combinations improved RGC survival above the levels achieved with individual factors alone (*P* > 0.05; Figure 3, A, C, E, and G).

### Long-term characterization of LIFR-induced regenerative effects after nerve injury

To determine whether the regenerative benefits conferred by LIFR overexpression endure beyond the acute post-injury phase, we extended our experimental endpoint to 8 weeks after ONC. Six experimental cohorts were included for comparison: AAV2-Ctrl; AAV2-LIFR; phosphatase and tensin homolog (*Pten*) deletion; AAV2-LIFR combined with *Pten* deletion; *Pten* deletion combined with the Zn^2+^ chelator TPEN; and the triple intervention group comprising *Pten* deletion, AAV2-LIFR, and intraocular TPEN.

At 8 weeks after nerve injury, a single treatment with AAV2-LIFR induced modest axon regeneration (Figure 4A), which was likely attributed to its failure to sustain robust RGC survival. Consistent with our short-term observations, AAV2-LIFR did not significantly improve RGC survival compared with AAV2-Ctrl at this time point (*P* > 0.05; Supplemental Figure 5, A and B). We further assessed visual function in the AAV2-Ctrl and AAV2-LIFR groups. Pattern electroretinography (pERG) recordings showed that AAV2-LIFR increased the N35-P50 amplitude to 1.7-fold of that in the AAV2-Ctrl group (*P* < 0.05; Figure 4, C and D). In contrast, the pERG P50-N95 amplitude remained unaltered after AAV2-LIFR administration (*P* > 0.05; Figure 4, C and E). Moreover, standalone AAV2-LIFR failed to produce significant improvements in pattern visual evoked potential (pVEP) or optomotor response (OMR) at 8 weeks after ONC (*P* > 0.05; Supplemental Figure 5, C–F).

Previous studies have validated that combined *Pten* deletion and intraocular TPEN treatment can durably promote RGC survival and axon regeneration (24). In line with these findings, we observed persistent and robust regenerative outcomes in the *Pten* deletion plus TPEN group. On this potent pro-regenerative background, supplementary AAV2-LIFR produced a modest, nonsignificant increase in axon regeneration, reaching 1.2-, 1.1-, 1.2-, and 1.8-fold the levels observed with *Pten* deletion plus TPEN at 250, 500, 1,000, and 2,000 μm distal to the injury site, respectively (*P* > 0.05; Figure 4, A and B). Additionally, AAV2-LIFR supplementation did not further increase RGC survival or alter the detected electrophysiological and behavioral functional outcomes in the context of *Pten* deletion combined with TPEN background (*P* > 0.05; Figure 4, C–E, and Supplemental Figure 5, A–F). These results suggest that AAV2-LIFR may confer an additional long-distance growth advantage in the *Pten* deletion plus TPEN background, though this promotional effect was subtle and did not reach statistical significance under our experimental conditions.

The most prominent supplementary beneficial effect of AAV2-LIFR was observed in the *Pten* deletion background. *Pten* deletion is a robust pro-regenerative manipulation that activates PI3K/AKT signaling (13, 25) and elicits a sustained regenerative response at 8 weeks after ONC (Figure 4A). Notably, the combination of AAV2-LIFR and *Pten* deletion exerted a synergistic pro-regenerative effect, markedly augmenting the regenerative capacity of *Pten* deletion alone. Axon regeneration in the combined group was increased to 1.8-fold at 250 μm, 1.9-fold at 500 μm, 1.8-fold at 1,000 μm, and 1.3-fold at 2,000 μm of the levels in the sole *Pten* deletion group (*P* < 0.0001 at 250 and 500 μm; *P* > 0.05 at 1,000 and 2,000 μm; Figure 4, A and B). This benefit was also validated by the retinal electrophysiological assay. In the *Pten* deletion background, AAV2-LIFR increased the pERG N35-P50 amplitude to 1.5-fold of that in the single *Pten* deletion group (*P* < 0.01; Figure 4, C and D). Despite no observable improvements in pVEP amplitude and OMR function after AAV2-LIFR supplementation in the *Pten* deletion model (*P* > 0.05; Supplemental Figure 5, C–F), our results confirm that LIFR overexpression can further potentiate *Pten* deletion–induced axon regeneration and partially restore RGC-associated retinal electrophysiological function at 8 weeks after ONC.

Longer-range anatomical analyses further characterized the degree of neural repair and regeneration. In both the single *Pten* deletion and combined treatment groups, axons labeled with cholera toxin subunit B (CTB) successfully extended to the optic chiasm, with only a small number of axons crossing the chiasmatic midline (*P* > 0.05; Supplemental Figure 5, G–I). However, none of the pro-regenerative intervention groups exhibited obvious CTB-positive axonal innervation in downstream visual relay regions, including the lateral geniculate nucleus and superior colliculus, in the examined pro-regenerative groups (Supplemental Figure 5J). Collectively, these long-term experimental data demonstrate that the combination of AAV2-LIFR and *Pten* deletion yields the most prominent supplementary therapeutic benefits. LIFR-targeted treatment can serve as an effective adjuvant strategy to enhance structural axonal regeneration and improve retinal electrophysiological function in conducive pro-regenerative settings. Nonetheless, full functional visual recovery and robust central target reinnervation require further optimized therapeutic interventions.

### LIFR knockdown suppresses optic nerve regeneration

To further investigate the contribution of LIFR to optic nerve regeneration, we knocked down expression of the receptor in RGCs of mature mice using an AAV2 expressing a targeted shRNA. After 2 weeks, IHC confirmed a reduction in LIFR intensity in RBPMS^+^ RGCs to background, that is, the level seen when the primary antibody was omitted during immunostaining (*P* < 0.05; Figure 5, A and B). Global LIFR knockdown was also investigated across the entire retina by immunoblotting and qPCR at 2 weeks after viral injection. Western blot analysis of whole retinal lysates revealed no statistically meaningful decline in total LIFR protein abundance compared with control retinas (*P* > 0.05; Supplemental Figure 6, A and B), whereas whole-retina qPCR showed a modest but significant 11% reduction in *Lifr* transcript levels after AAV2-shLIFR delivery (*P* < 0.05; Supplemental Figure 6C). This transcriptional repression aligned with quantitative fluorescence measurements performed in RBPMS^+^ RGCs. AAV2-shLIFR reduced zymosan-induced axon regeneration to the baseline level seen after ONC alone (*P* < 0.0001; Figure 5, C and D), and somewhat unexpectedly, decreased zymosan-induced RGC survival to the baseline level (*P* < 0.001; Figure 5, C and E). To explore the importance of LIFR more generally, we also tested the effect of AAV2-shLIFR on regeneration induced by knockdown of PTEN. AAV2-shLIFR fully abolished both axon regeneration and the neuroprotective effect of PTEN knockdown (*P* < 0.001; Figure 5, F–H).

miRNAs are small noncoding RNAs that play important regulatory roles by targeting mRNAs for cleavage or translational repression (26). miR-21 has been reported to interact with *Lifr* mRNA directly (27, 28), which we verified by showing that miR-21 binds to the 3′ UTR of *Lifr* mRNA (dual-luciferase reporter assay; Supplemental Figure 6, D and E). We found that miR-21 was highly expressed in RBPMS^+^ RGCs using FISH (Supplemental Figure 6F). We next constructed an AAV2 expressing miR-21 antisense sequences (AAV2-miR-21-sponge) to sequester miR-21 away from its endogenous targets. Like LIFR overexpression, AAV2-miR-21-sponge enhanced axon regeneration 4.5-fold over baseline (*P* < 0.0001 compared with AAV2-Ctrl-sponge; Supplemental Figure 6, G and H), with no significant impact on RGC survival (*P* > 0.05; Supplemental Figure 6, G and I). However, genetic knockdown of LIFR via intravitreal AAV2-shLIFR administration 3 weeks prior to ONC completely abrogated the pro-regenerative effect induced by intravitreal AAV2-miR-21-sponge delivered 2 weeks before ONC (*P* < 0.0001; Figure 5, I and J). LIFR knockdown did not significantly alter RGC survival in the AAV2-miR-21-sponge–treated group (*P* > 0.05; Figure 5, I and K) These findings further validate the role of LIFR as an essential regulator of RGC axonal regeneration.

### Transcriptomic analyses

To investigate the molecular consequences of LIFR overexpression, RGCs from *Vglut2*-tdTomato mice were FACS-isolated 2 weeks after intraocular injection of AAV2-LIFR or AAV2-Ctrl, and then analyzed by bulk RNA-seq. AAV2-LIFR had pronounced transcriptional effects on adult RGCs, as indicated by principal component analysis (PCA; Supplemental Figure 7A) and the vast number of differentially expressed genes (DEGs) (4,020 DEGs, *q* < 0.05, |log_2_FC|>1, 2,112 upregulated and 1,908 downregulated, AAV2-LIFR vs. AAV2-Ctrl; Figure 6A and Supplemental Figure 7C). AAV2-LIFR upregulated genes encoding many growth factors (e.g., *Egf*, *Ngf*, *Igf2*, *Ccl5*) and receptors (*Igf1r*, *Igf2r*), along with the expected increase in *Lifr*, while downregulating the expression of genes associated with inhibition of regeneration (*Rtn4r* [Nogo receptor, NgR], *Rtn4rl2* [NgR2], *Lingo1*, *Cspg5*), growth cone collapse (*Sema6c*, *Sema4f)*, and cell death (*Nlrp1a*, *Faf1*) (Figure 6B). qPCR validation performed on FACS-isolated RGCs confirmed significant expression alteration of representative RNA-seq hits, including *Lifr* (*P* < 0.05), *Ngf* (*P* < 0.01), and *Faf1* (*P* < 0.001) (Supplemental Figure 7, E–G). Gene Ontology (GO) enrichment analysis revealed that LIFR overexpression upregulated groups of genes relevant to positive regulation of the MAPK cascade, positive responses to cytokine stimulus, cytokine receptor activity, positive regulation of axon regeneration, and regulation of receptor signaling pathways via JAK/STAT and downregulated groups of genes associated with autophagic cell death, programmed necrotic cell death, intrinsic apoptotic signaling, and negative regulation of axonogenesis, axon extension, and regeneration (Figure 6C).

In complementary studies, we explored the transcriptional effect of knocking down LIFR (via AAV2-shLIFR) in RGCs, again using bulk RNA-seq. As with LIFR overexpression, LIFR knockdown had robust transcriptional effects as revealed by PCA, heatmap analysis (Supplemental Figure 7B and Figure 6D), and the large number of DEGs (4,708 DEGs, *q* < 0.05, |log_2_FC|>1, 2,065 upregulated and 2,643 downregulated, AAV2-shLIFR vs. AAV2-shScramble; Figure 6D and Supplemental Figure 7D). In a reversal of the effects of LIFR overexpression, LIFR knockdown enhanced the expression of myelin-associated inhibitors of axon growth and their receptors (*Rtn4* [Nogo], *Rtn4r* [NgR], and *Mbp*), while downregulating anti-apoptotic genes (*Bcl2a1a*, *Bcl2a1b*, *Bmpr1b*) and genes encoding growth factors, receptors, and their effectors (*Igf1*, *Isl2*, *Il6ra*, *Nefl*, *Nefh*, *Akt2*, and *Jak3*) (Figure 6E). At the same time, GO enrichment analysis showed that LIFR knockdown led to the upregulation of groups associated with programmed cell death, damaged DNA binding, and negative regulation of axonogenesis and axon regeneration, along with downregulation of terms relevant to the positive regulation of MAPK cascade, cellular response to IGF and NGF (and LIF), cytokine-mediated signaling pathways, positive regulation of axon regeneration, and RGC axon guidance (Supplemental Figure 7H).

### Supporting evidence for LIFR regulation of signal transduction pathways

We further investigated the role of LIFR in neuroprotective and pro-regenerative signaling pathways by IHC. As before, we manipulated levels of LIFR expression by intraocular injections of either AAV2-LIFR or AAV2-shLIFR, and then evaluated levels of phosphorylated (active) ERK (pERK), phosphorylated protein kinase B (pAKT), phosphorylated ribosomal protein S6 (pS6), and phosphorylated STAT 3 (pSTAT3) in RBPMS^+^ RGCs 2 weeks later using appropriate antibodies. In conformity with the transcriptomic results, LIFR overexpression increased pERK levels in RGCs (*P* < 0.01 compared with the AAV2-Ctrl group; Figure 7, A and C), whereas LIFR knockdown reduced pERK levels in RGCs (by 33.7%, *P* < 0.01 compared with AAV2-shScramble; Figure 7, A and C). AAV2-shLIFR also reduced levels of pAKT and pS6, 2 markers of mTOR pathway activation, by 45.7% and 47.0%, respectively (all *P* < 0.001 compared with the AAV2-shScramble group; Figure 7, A, D, and E). LIFR overexpression also seemed to reduce pAKT and pS6 levels, though this did not attain statistical significance (*P* > 0.05; Figure 7, A, D, and E). No significant differences were observed for pSTAT3 levels in RGCs as a result of LIFR overexpression or knockdown (*P* > 0.05; Figure 7, B and F).

To compare the effects of LIFR overexpression with those of LIF itself, we also evaluated expression of the above signal molecules by IHC 3 days after intraocular injections of recombinant LIF protein (1 μg/eye). Intraocular LIF did not alter pERK, pAKT, or pS6 expression (compared with BSA control, *P* > 0.05; Figure 7, A and C–E), whereas, as expected, it increased pSTAT3 levels (*P* < 0.001; Figure 7, B and F). This finding suggests the possibility that LIFR overexpression and LIF might activate distinct downstream signaling pathways, although the mechanisms involved are unclear.

Given that LIFR manipulation alters pERK levels and MAPK-related transcriptional signatures, we tested whether activation of MAPK signaling could rescue the regenerative deficits induced by LIFR knockdown. BRAF is an upstream activator of the MEK/ERK cascade, and activated BRAF signaling is an established driver of CNS axon regeneration (29). In the context of LIFR depletion via AAV2-shLIFR, AAV2-mediated BRAF overexpression largely restored axonal regeneration, increasing the number of regenerating axons 62.4-fold (*P* < 0.001; Supplemental Figure 8, A and B) distal to the injury site relative to AAV2-shLIFR alone. In addition, AAV2-BRAF increased RGC survival 1.3-fold compared with the AAV2-shLIFR group (*P* < 0.01; Supplemental Figure 8, A and C). Collectively, these data indicate that MAPK pathway activation represents a major component of LIFR-dependent optic nerve regeneration, and BRAF overexpression can only partially reverse the impaired regenerative phenotype caused by LIFR knockdown.

### The intracellular domain of LIFR is sufficient to promote optic nerve regeneration

Because our experiments showed that the benefits of LIFR overexpression have limited dependence on its cognate ligands, we investigated whether a truncated form of the receptor lacking extracellular ligand-binding sites is sufficient to stimulate regeneration. For this, we designed an AAV vector expressing a truncated LIFR (AAV2-LIFRΔ) that includes only the signal peptide, transmembrane, and intracellular domains. Two weeks after AAV transduction, anti-FLAG immunoblotting of whole retinal lysates readily detected full-length LIFR protein in retinas receiving AAV2-LIFR, whereas no corresponding band was observed in the AAV2-LIFRΔ group (Supplemental Figure 9A). Quantification further verified markedly diminished FLAG signals in AAV2-LIFRΔ–treated retinas relative to retinas transduced with full-length AAV2-LIFR (*P* < 0.01; Supplemental Figure 9B), confirming that the truncated AAV2-LIFRΔ construct fails to produce measurable full-length LIFR protein. Two weeks after intraocular injection of AAV2-LIFRΔ, we injured the optic nerve and evaluated regeneration after another 2 weeks. AAV2-LIFRΔ elevated axon regeneration 3.2-fold compared with AAV2-Ctrl (*P* < 0.001; Figure 8, A and B), which was only slightly lower than overexpression of the full-length receptor (26.0% lower compared with AAV2-LIFR: *P* > 0.05; Figure 8, A and B). Like AAV2-LIFR, AAV2-LIFRΔ did not affect RGC survival (*P* > 0.05; Figure 8, A and C). These results indicate that extracellular domain–truncated LIFR retains substantial pro-regenerative capacity, highlighting the critical functional contribution of LIFR’s transmembrane and intracellular domains and the independence of LIFR-mediated regeneration of its canonical ligand.

Prior structure-function studies of LIFR have shown that cytoplasmic regions of LIFR and gp130 act cooperatively to mediate downstream signal transduction (30), with the Box1 motif within the LIFRα cytoplasmic tail serving as a functionally indispensable region (31). In addition, modular tyrosine-based motifs in cytokine receptor complexes, including those containing LIFR, are known to recruit STAT proteins and other downstream effectors to fine-tune signaling cascades (32). Therefore, we constructed 2 full-length LIFR mutant vectors: one with a specific deletion of the Box1 motif (AAV2-LIFR-ΔBox1) and another carrying a tyrosine-to-phenylalanine point mutation at the Y969 residue (AAV2-LIFR-Y969F). We found that both mutants exhibited substantially impaired pro-regenerative activity relative to WT LIFR. The number of regenerating axons elicited by each mutant was reduced to 44% of that observed in the AAV2-LIFR group. No significant difference was detected between the AAV2-LIFR-ΔBox1 and AAV2-LIFR-Y969F mutant groups (*P* > 0.05; Figure 8, D and E). Meanwhile, RGC survival in both mutant groups remained comparable to that observed in the AAV2-LIFR group, with no significant difference between each mutant group and the AAV2-LIFR baseline (*P* > 0.05; Figure 8, D and F). These results indicate that Box1 and Y969 are required to mediate the maximal pro-regenerative output of LIFR; nevertheless, disruption of either motif alone fails to fully ablate LIFR-dependent axonal regenerative capacity.

We also explored the role of LIFR on different RGC subtypes by immunostaining for SMI32 (against neurofilament heavy chain for αRGCs), cocaine- and amphetamine-regulated transcript (CART) (for on-off direction-selective RGCs, ooDSGCs), and melanopsin (intrinsically photosensitive RGCs, ipRGCs). IHC verified that all 3 markers colocalized with the pan-RGC marker, RBPMS (Supplemental Figure 9C). Again, AAV2-LIFRΔ was intraocularly injected 2 weeks before nerve injury, and the survival of the 3 RGC subtypes was assessed after another 1 or 2 weeks. Despite the absence of an overall effect on RGC survival, AAV2-LIFRΔ strongly increased ipRGC survival (by 47.6% at 1 week and 48.3% at 2 weeks after injury, respectively, both *P* < 0.05; Supplemental Figure 9, D and E), decreased the survival of ooDSGCs (by 40.4% at 1 week and 41.9% at 2 weeks after injury; *P* < 0.01, *P* < 0.05, respectively; Supplemental Figure 9, D and F), and did not affect the survival of αRGCs (*P* > 0.05; Supplemental Figure 9, D and G). Thus, LIFRΔ appears to alter RGC survival in a subtype-specific way. Figure 9 schematically illustrates our major findings.

Finally, we characterized the expression of LIFR in sections of a postmortem human retina from a 17-year-old male. IHC revealed abundant LIFR expression in RBPMS^+^ RGCs in the human retina (Figure 10).

## Discussion

LIFR is a 190 kDa protein containing an extracellular ligand-binding domain, a transmembrane domain anchoring the receptor in the cell membrane, and an intracellular domain involved in signal transduction (33). Like CNTFRα (34), LIFR functions as both a soluble and a membrane-bound receptor that forms a complex with gp130 (35), a receptor subunit that is shared by other cytokine receptors in the IL-6 family (36, 37). LIF binds with high affinity to the receptor complex, forming a heterodimeric complex that activates multiple signal transduction pathways (38). However, our RNA-seq analysis of adult RGCs revealed that LIFR regulates signal transduction pathways and gene expression with limited dependence on LIF. Increasing LIFR expression is sufficient to increase the activity of the MAPK pathway and expression of other growth factors, receptors, and groups of genes related to axonogenesis, growth cone formation, cytoskeletal organization, and response to cytokines and to myelin-associated inhibitors of axon growth with limited dependence on ligand availability, whereas LIFR knockdown decreases the activity of both the MAPK and AKT signaling pathways, with transcriptional effects that are largely opposite to those of LIFR overexpression.

In addition to LIFR, adult RGCs express other receptors that participate in controlling axon regeneration, including ArmC10 (8), CCR5 (14), CXCR4 (7), and IGF1R (11). Brain-derived neurotrophic factor (BDNF) antagonizes zymosan-induced optic nerve regeneration (39), although virally mediated expression of BDNF and its receptor, TrkB, enhances RGC survival (40), as reported by us and others. However, to our knowledge, LIFR is unique in its central role in enabling optic nerve regeneration with limited dependence on ligand availability.

During CNS development, LIFR is essential for modulating the differentiation and maturation of neuronal and non-neuronal cells (41–43) and can alleviate demyelination by enhancing oligodendrocyte survival after CNS injury (44, 45). However, whether the ligand, LIF, and related neurokine CNTF are sufficient to induce optic nerve regeneration under normal circumstances is a question. In agreement with several other studies, we found that, whereas CNTF by itself has little effect on optic nerve regeneration (14, 39, 46, 47), CNTF gene therapy has robust effects (38, 48–50) as a consequence of amplifying a virally induced immune response and elevating levels of chemokine CCL5, the proximate inducer of axon regeneration (14). In addition, unlike the profound loss of regeneration found when LIFR is deleted in RGCs, deletion of the CNTF receptor CNTFRα still allows RGCs to regenerate axons, including in response to AAV2-CNTF (14). Regarding the ligand LIF, whereas one group has reported positive effects on optic nerve regeneration (16, 51), we found little or no effect of LIF in the absence of receptor overexpression. Nonetheless, because zymosan-induced intraocular inflammation elevates levels of both LIF in neutrophils and LIFR in RGCs, it is possible that immune cell–derived LIF may supplement the effects of Ocm and SDF1 in mediating inflammation-induced regeneration. Although blocking the effects of Ocm and SDF1 fully abrogates zymosan-induced regeneration (7), a noncritical but supportive role of LIF cannot be excluded. Finally, it is important to emphasize that, although prior studies have examined the role of LIF, none has previously examined the distinct ligand-independent role of LIFR per se, to our knowledge.

In parallel to the developmental decline of RGCs’ growth capacity (52, 53), *Lifr* mRNA levels decrease with maturation, suggesting that LIFR may be one of the contributory factors in the decline of RGCs’ intrinsic growth potential. In addition, like *Pten*, *Socs3*, *Myc*, and other transcription factors, our gain- and loss-of-function studies point to LIFR being a critical regulator of optic nerve regeneration. Finally, our finding that LIFR regulates adult RGCs’ responsiveness to the cognate ligand, unrelated trophic factors, and other therapies suggests that combinatorial therapies that include LIFR may maximize pro-regenerative outcomes in optic neuropathies and perhaps in other CNS neurodegenerative diseases, bringing us closer to therapeutic translation.

## Methods

### Sex as a biological variable

Our study examined male and female animals, and similar findings are reported for both sexes.

### Animals and surgeries

The experiments used female and male C57BL/6J mice (Hunan SJA Laboratory), B6-G/R reporter mice (GemPharmatech, strain T006163), *Vglut2*-Cre mice (The Jackson Laboratory, strain 016963), and *Pten^fl/fl^* mice (Cyagen, strain S-CKO-04509). B6-G/R reporter mice were crossed with *Vglut2*-Cre mice to generate *Vglut2*-tdTomato mice for RGC labeling. Surgeries for ONC and intravitreal injection were carried out under general anesthesia as described previously (54–56).

Reagents that were injected into the vitreous chamber included zymosan (12.5 μg/μL, 3 μL/eye, Sigma-Aldrich, Z4250), a yeast-derived glucan; recombinant mouse LIF protein (1 μg/eye, PeproTech, 250-02); recombinant mouse IGF protein (1 μg/eye, PeproTech, 250-19); mouse SDF1 protein (0.9 μg/eye, PeproTech, 250-20A); mouse Ocm protein (90 ng/eye; LSbio, LS-G57232); CPT-cAMP (50 μM, 3 μL/eye; Sigma-Aldrich, C3912); LIF neutralizing antibody (400 ng/mL, 3 μL/eye, R&D Systems, ab449NA); CTF1 neutralizing antibody (400 ng/mL, 3 μL/eye, R&D Systems, AF438SP); OSM neutralizing antibody (3,000 ng/mL, 3 μL/eye, R&D Systems, AF495NA); mouse IgG isotype control (Abcam, ab37355); N,N,N′,N′-tetrakis (2-pyridylmethyl) ethylenediamine (TPEN; 100 μM, 3 μL/eye, Sigma-Aldrich, P4413).

AAV vectors used in this study included AAV2 expressing full-length LIFR or negative control sequence (AAV2-LIFR, AAV2-Ctrl); AAV2 expressing shRNA targeting *Lifr*, *Pten,* or scramble sequence (AAV2-shLIFR, AAV2-shPTEN, AAV2-shScramble) and AAV2 expressing antisense sequences for miR-21 (AAV2-miR-21-sponge) or negative control (AAV2-Ctrl-sponge, GenePharma Co., Ltd); AAV2-Cre for conditional *Pten* deletion in *Pten^fl/fl^* mice; AAV2 expressing mouse BRAF (AAV2-BRAF); AAV2 expressing a truncated LIFR lacking the extracellular domain (AAV2-LIFRΔ); and AAV2 expressing full-length LIFR mutants lacking the Box1 motif or carrying a tyrosine-to-phenylalanine substitution at Y969 (AAV2-LIFR-ΔBox1 and AAV2-LIFR-Y969F).

All AAV vectors utilized in this study were CAG promoter-driven and were delivered intravitreally at 3 μL/eye. Viral titers varied across different constructs, ranging from 2 × 10^12^ to 1 × 10^13^ vg/mL. To ensure viral transduction and sufficient expression of target transgenes or shRNAs, all AAV injections were performed 2–3 weeks prior to ONC according to the experimental design. Nonviral agents were administered immediately after nerve injury. CTB (2 μg/μL, Invitrogen, C22843) was intraocularly injected 2 days before perfusion of mice to trace regenerated axons.

### Human tissue collection

A postmortem human eye was collected from a 17-year-old male donor without a known history of eye disease after removing the cornea with approval from the Ethics Committee of the Second Xiangya Hospital. All processes in this study followed the tenets of the Declaration of Helsinki.

### Separation and identification of neutrophils in vitreous

To isolate neutrophils in the vitreous at different time points after intraocular zymosan, the vitreous was extracted into 500 μL PBS and incubated with anti-Ly6G MicroBeads (Miltenyi Biotec,130-120-337) after washing (following the manufacturer’s protocol). Ly6G^+^ cells were captured by magnetic-activated cell sorting. To evaluate the purity of separated cells, cells were incubated with Gr-1 antibody conjugated with FITC (Miltenyi Biotec, 130-102-338z), and purity was measured by flow cytometry using BD Biosciences FACSCanto II. To characterize the morphology of isolated Ly6G^+^ cells, Giemsa staining was performed using Fast Giemsa Stain kit (Solarbio, G4640) according to the manufacturer’s protocol after cell fixation. Cells were observed and imaged under a Leica CTR4000 microscope.

### FACS isolation of RGCs

*Vglut2*-tdTomato mice received an intravitreal injection of AAV2-LIFR, AAV2-Ctrl, AAV2-shLIFR, or AAV2-shScramble. Mice with different treatments were euthanized 14 days after viral vector injection to ensure the expression of encoded sequences. Retinas were carefully dissected and dissociated by trituration after incubation with papain (Sangon Biotech, A003124-0100) for 12 minutes at 37°C. TdTomato^+^ cells were isolated by FACS using the BD Biosciences FACSAria III instrument. Four retinas were pooled per replicate, yielding approximately 20,000 cells.

### qPCR

Total RNA was extracted from RGCs, ly6G^+^ neutrophils, or retina using TRIzol (Invitrogen, 15596026) following the manufacturer’s instructions. cDNA was synthesized with RevertAid First Strand cDNA Synthesis kit (Thermo Fisher Scientific, K1621). qPCR was performed using Maxima SYBR Green/ROX qPCR Master Mix (Thermo Fisher Scientific, K0221) and the following primers: Lifr-F: ATCGATGTGCAGTCCATGTATC, Lifr-R: ATTCCAAGTGTTTACATTGGCC, Lif-F: GCTACTATAGACGTCATGAGGG, Lif-R: CAACCCAACTTTTTCCTTTGGA, Gapdh-F: GGTTGTCTCCTGCGACTTCA, and Gapdh-R: TGGTCCAGGGTTTCTTACTCC.

Relative expression levels were first normalized to Gapdh and then to the corresponding control group. Because neutrophils are uncommon in the vitreous without intraocular zymosan, absolute quantification by qPCR was used to measure the exact copy number of *Lif* mRNA in neutrophils using external standards. For absolute quantification of *Lif* transcripts, external standard curves were generated via 10-fold serial dilutions of recombinant plasmid DNA carrying a mouse *Lif* cDNA fragment, with concentrations spanning 10^2^ to 10^10^ copies/μL. Ct values were converted to *Lif* mRNA copy numbers using the standard curve.

### Evaluation of optic nerve regeneration and RGC survival

Animals were perfused transcardially with saline and 4% PFA (Biosharp, BL539A). Optic nerves and retinas were dissected and postfixed in 4% PFA for 1 hour at room temperature. Postfixed nerves were embedded into OCT compound (Sakura, 4583) and cryostat-sectioned longitudinally at 14 μm after transferring to 30% sucrose solution at 4°C overnight. Axons labeled by Alexa Fluor 555–conjugated CTB (Invitrogen, C22843) were counted in at least 3 sections per case at 500 μm distal to the crush site, as described previously (17, 54, 57). For the 8-week experimental cohort, CTB-labeled axons were further quantified at 1,000 and 2,000 μm distal to the crush site. The total number of regenerating axons per nerve at each measured distance was calculated using the following formula:

Σa _d_ = π*r*² × (average axons/mm)/*t*,

where *r* is the optic nerve radius, and *t* is the section thickness (57). Counts from serial sections were averaged to yield a single representative measurement for each animal. Whole-mounted retinas were immunostained for βIII-tubulin (TUJ1, 1:500, Abcam, ab18207) to evaluate RGC survival. TUJ1-immunopositive RGCs were quantified across 8 predefined fields per retina: 2 fields were sampled within each of the 4 retinal quadrants, as described previously with minor modifications (54, 58). TUJ1-positive somas in the ganglion cell layer were counted and normalized to the imaging area to calculate RGC density (cells/mm^2^). RGC densities from the 8 fields were averaged for each retina. For RGC subtype analysis, retinal flat mounts were immunostained for CART (1:2,000, Phoenix Pharmaceuticals, H-003-62), Melanopsin (1:4,000, ATS, ATS-AB-N39), and SMI32 (1:300, BioLegend, 801701) to visualize ooDSGCs, ipRGCs, and αRGCs, respectively, which were evaluated using the identical 8-field sampling strategy and normalized to cell density (cells/mm^2^) (54). Images were taken using Axio Imager M2+ApoTome.3 microscope (Zeiss).

### Adult dissociated retina culture

AAV2-LIFR or AAV2-Ctrl was intraocularly injected into *Vglut2*-tdTomato mice 2 weeks before culture. Retinas from *Vglut2*-tdTomato mice were used to visualize RGCs in the dissociated retina culture system. The procedure for adult retinal culture has been described previously (9, 55). Briefly, retinas were dissected from adult *Vglut2*-tdTomato mice and dissociated after incubating with papain (Sangon Biotech, A003124-0100). Retinal cells were cultured in F12 medium supplemented with B-27 (Gibco, 17504044), l-glutamine (Gibco, 25030081), and penicillin-streptomycin (Gibco, 15140122). Additional recombinant LIF protein (100 or 300 ng/mL, PeproTech, 250-02) was added into the culture medium to test the effect of LIF on adult RGCs’ neurite outgrowth with or without intraocular AAV2-LIFR. Three days after cell plating, neurite outgrowth and RGC survival were quantified in 9 preselected fields per well in 96-well plates. Images were acquired under identical settings across all experimental groups. Neurite outgrowth was quantified as the proportion of tdTomato-positive RGCs bearing neurites, whereas RGC survival was quantified as the total number of tdTomato-positive RGCs across the 9 fields per well. Each condition included 4–5 replicates.

### Preparation and immunostaining of retinal cross sections

Mice were perfused transcardially with saline followed by 4% PFA. The eyes were dissected and postfixed in 4% PFA for 2 hours at room temperature. The eyeballs were embedded in OCT compound and cryostat-sectioned at 14 μm after transferring into 30% sucrose overnight at 4°C. For retinal cross-section analysis, a minimum of 3 nonconsecutive sections were randomly selected from the seria l section series of each eye for analysis. Only sections displaying intact retinal architecture and uninterrupted retinal layers spanning from the central to peripheral retina were included for subsequent analysis. Sections were blocked with 5% BSA containing 0.5% Triton X-100 (DingGuo, DH351-4). The primary antibodies used in this study included anti-RBPMS (1:400, MilliporeSigma, ABN1376), anti-βIII-tubulin (1:500, Abcam, ab18207), anti-LIFR (1:900, Abcam, ab101228), monoclonal Gr1 (1:500, LSBio, LS-C112469), anti-pS6 (Ser240/244) (1:800; Cell Signaling Technology, 5364), anti-pERK1/2 (Thr202/Tyr204) (1:200; Cell Signaling Technology, 4370), anti-pSTAT3 (1:500; Abcam,ab76315), and anti-pAKT (T308) (1:100; Abcam, ab38449). Secondary antibodies included Alexa Fluor 488 anti-mouse IgG (1:500; Abcam, ab150113), Alexa Fluor 488 anti-rabbit IgG (1:500; Abcam, ab150077), Alexa Fluor 555 anti-mouse IgG (1:500; Abcam, ab150118), Alexa Fluor 555 anti-rabbit IgG (1:500; Abcam, ab150078), Alexa Fluor 647 anti-mouse IgG (1:500; Abcam, ab150115), and Alexa Fluor 647 anti-rabbit IgG (1:500; Abcam, ab150083). Sections were counterstained with DAPI and imaged using an Axio Imager M2 equipped with ApoTome.3 (Zeiss).

### Western blotting

Retinal tissues were lysed on ice in RIPA buffer (Beyotime, P0013B) supplemented with PMSF (Biosharp, BL507A) and PhosSTOP (Roche, 04906837001). Protein concentrations were determined using a BCA protein assay kit (Beyotime, P0010), and equal amounts of protein were separated by SDS-PAGE and transferred onto PVDF membranes. After blocking, membranes were incubated overnight at 4°C with anti-LIFR (1 μg/mL; Abcam, ab101228) or anti-FLAG (1:1,000; Sigma-Aldrich, F1804), followed by HRP-conjugated goat anti-rabbit IgG (Beyotime, A0208) or goat anti-mouse IgG (Beyotime, A0216), as appropriate. Protein bands were visualized using an enhanced chemiluminescence detection system.

### HEK293T cell line culture and plasmid transfection

HEK293T cells (ATCC, CRL-3216) were cultured in DMEM (Gibco, c11995500bt) supplemented with 10% FBS (Bovogen, SFBS) at 37°C. Cells were seeded in 12-well plates at a density of 5 × 10^5^ cells per well 24 hours before transfection. Plasmid transfections were performed using Lipofectamine 3000 (Invitrogen, 3000015) according to the manufacturer’s protocol.

### Dual-luciferase reporter assay

The miR-21–binding sequence in the WT 3′ UTR of *Lifr* was inserted downstream of the luciferase reporter gene in the luciferase reporter vector. The miR-21 binding site mutant of 3′-UTR *Lifr* was generated and inserted into another luciferase reporter vector. A vector containing WT or mutant binding sequence, together with a vector expressing miR-21 (miR-21 mimics) were transfected into HEK293T cells. Luciferase activity was analyzed by a dual-luciferase reporter assay system (Promega, E2910) 48 hours after transfection.

### Functional testing

#### pERG.

For pERG recordings, mice were anesthetized, and pupils were dilated with 0.5% tropicamide (Santen Pharmaceutical Co., Ltd.). A ground electrode was inserted at the base of the tail, a reference electrode was placed subcutaneously between the orbits, and gold-ring electrodes were positioned on each cornea. The contralateral eye was fully light-blocked during recording. Pattern stimuli comprised contrast-reversal horizontal black-and-white square-wave gratings with a spatial frequency of 0.125 cycles/degree and a temporal frequency of 1 Hz. Signals from 200 trials were averaged within the Multifocal Visual Electrophysiological System (RetiMINER System, Chongqing IRC Medical Equipment Co., Ltd.). N35-P50 and P50-N95 peak amplitudes were quantified from averaged composite waveforms.

#### pVEP.

For pVEP recordings, anesthetized mice were fixed facing the pattern stimulus monitor. The active recording electrode was implanted subcutaneously above the primary visual cortex; the reference electrode was placed subcutaneously between the ears, and the ground electrode was placed at the tail. Pattern-reversal stimuli were delivered using horizontal black-and-white square-wave gratings with a spatial frequency of 0.125 cycles/degree and a temporal frequency of 1 Hz. Signals were averaged across 200 trials using the RetiMINER System. The N75-P100 and P100-N135 amplitudes were measured from the averaged pVEP waveforms.

#### OMR.

OMR testing was performed using the OptoTrack system (XR-OT101, Shanghai XinRuan Information Technology Co., Ltd.). Mice were placed on a raised central platform with unrestricted head and body movement. Vertical sinusoidal gratings were projected onto 4 surrounding LCD screens to form a virtual rotating cylinder, which randomly rotated clockwise or counterclockwise at 12°/s. A high-speed camera continuously tracked spontaneous head-tracking movements. Spatial frequencies of 0.05, 0.1, 0.2, and 0.3 cycles/degree were sequentially tested. OMR values were automatically analyzed using OptoTrack v2.0.0.1 software.

### RNA-seq library construction and sequencing

Total RNA was extracted from FACS-sorted RGCs using Trizol reagent (Invitrogen, 15596026) according to the manufacturer’s instructions. The purity and concentration of the RNA were measured using the NanoDrop 2000 spectrophotometer (Thermo Fisher Scientific). RNA integrity was assessed using the Agilent 2100 Bioanalyzer. The RNA-seq library was prepared using the VAHTS Universal V5 RNA-seq Library Prep kit (Vazyme Biotech) following the manufacturer’s protocol. Transcriptome sequencing and subsequent data analysis were conducted by OE Biotech Co., Ltd.

### RNA-seq data processing

Libraries were sequenced on an Illumina NovaSeq 6000 platform, and 150 bp paired-end reads were generated. Raw reads of FASTQ format were processed using fastp (59) to remove low-quality reads and obtain clean reads for subsequent analysis. Clean reads were then aligned to the Genome Database using HISAT2 (60). FPKM of each gene was calculated, and the read counts of each gene were obtained by HTSeq-count (61). PCA was performed using R (v3.2.0) to evaluate the biological duplication of samples. Differential expression analysis was performed using DESeq2 (62). Genes with *q* less than 0.05 and |log_2_FC| greater than 1 were identified as DEGs. Hierarchical clustering of DEGs was conducted to illustrate expression patterns across different groups and samples. Based on the hypergeometric distribution, GO enrichment analysis (63) of DEGs was performed to identify enriched terms using R (v3.2.0).

### Statistics

Values are presented as mean ± SEM. Comparisons between 2 groups were performed using unpaired 2-tailed Student’s *t* tests. Comparisons among 3 or more groups were performed using 1-way ANOVA followed by Tukey’s multiple-comparison test. Two-way ANOVA followed by Šidák’s or Tukey’s multiple-comparison test, as appropiate, was applied for datasets containing 2 independent variables. All statistical analyses were performed using GraphPad Prism 9. *P* values less than 0.05 were considered statistically significant.

### Study approval

All animal experiments were conducted at the Second Xiangya Hospital of Central South University and approved by the IACUC (approval 2022242). Human retinal tissues were obtained with approval from the Ethics Committee of the Second Xiangya Hospital (approval 0333). All procedures adhered to the tenets of the Declaration of Helsinki. Written informed consent was obtained for the donation and research use of the human tissue, and the records of consent have been retained by the corresponding author.

### Data availability

All Supporting data values associated with the main manuscript and supplemental materials are provided in the Supporting Data Values file. The RNA-seq data were deposited in NCBI’s Gene Expression Omnibus (GSE274285).

## Author contributions

QJ performed most animal experiments included in the paper and drafted the manuscript. CW carried out cell culture experiments. YR carried out FISH and dual-luciferase reporter assay experiments. PD and KT collected the human tissue. XD, BC, and CX provided technical assistance. LB provided essential guidance for the project, reviewed all data, and assisted in writing. KL performed additional experiments, analyzed data, and contributed to manuscript revision. JL and NW provided ideas for additional experiments. BJ supervised many of the studies. LX supervised and oversaw the project and assisted in writing. All authors reviewed the manuscript.

## Conflict of interest

The authors have declared that no conflict of interest exists.

## Funding support

- National Natural Science Foundation of China (82070967 and 81770930 to BJ; 82201189 to LX).
- Hunan Provincial Key Research and Development Program (2023SK2029 to BJ; 2024DK2003 to KL).
- Natural Science Foundation for Excellent Young Scholars (Category A) of Hunan Province (2025JJ20080 to LX).

## Supplementary Material

Supplemental data

Unedited blot and gel images

Supporting data values

## Figures and Tables

**Figure 1:**
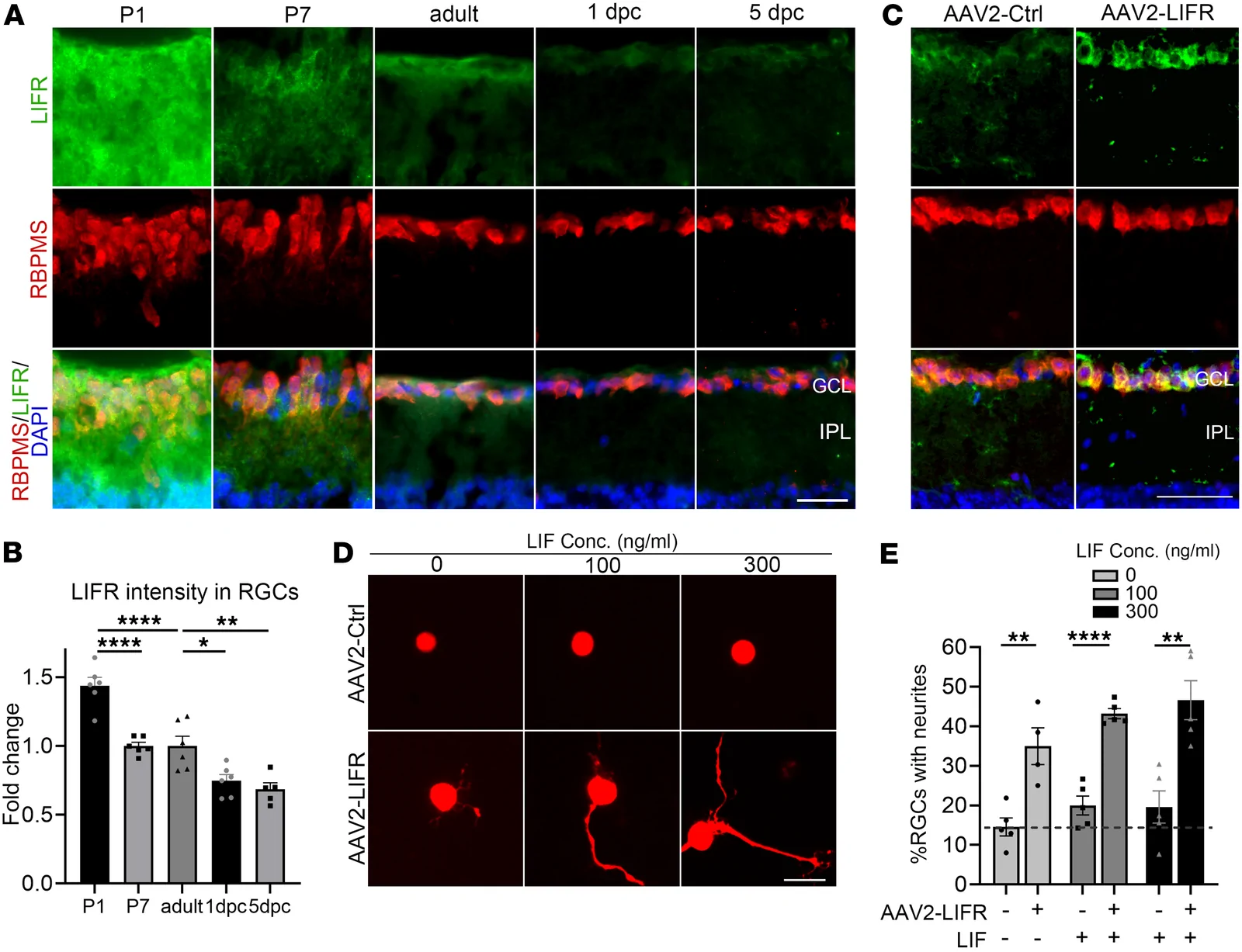
LIFR expression in RGCs. (**A**) Retinal cross sections from P1, P7, and mature (6 weeks) mice; and 1, 3, and 5 days after optical nerve crush (dpc) immunostained for LIFR (green), RBPMS (red), and DAPI (blue). GCL, ganglion cell layer; IPL, inner plexiform layer. Scale bar: 30 μm. This experiment was conducted once. (**B**) Quantification of LIFR intensity in RGCs from mice at time points shown in **A**. Statistical significance was determined by 1-way ANOVA followed by Tukey’s multiple-comparison test (*P < 0.05, **P < 0.01, ****P < 0.0001; *n* = 5–6 eyes per group). (**C**) Retinal cross sections from mice 2 weeks after AAV2-Ctrl or AAV2-LIFR injection immunostained with antibodies to LIFR (green), RBPMS (red), and DAPI (blue). Scale bar: 30 μm. This experiment was conducted once. (**D**) Representative images showing neurite outgrowth in cultured RGCs (red) from *Vglut2*-tdTomato mice with indicated treatments at day 3. Scale bar: 50 μm. This experiment was conducted once. (**E**) Quantitation of neurite outgrowth in RGCs. Statistical significance was determined by unpaired 2-tailed *t* test (**P < 0.01, ****P < 0.0001; *n* = 4–5 wells per group). Data represent mean ± SEM.

**Figure 2:**
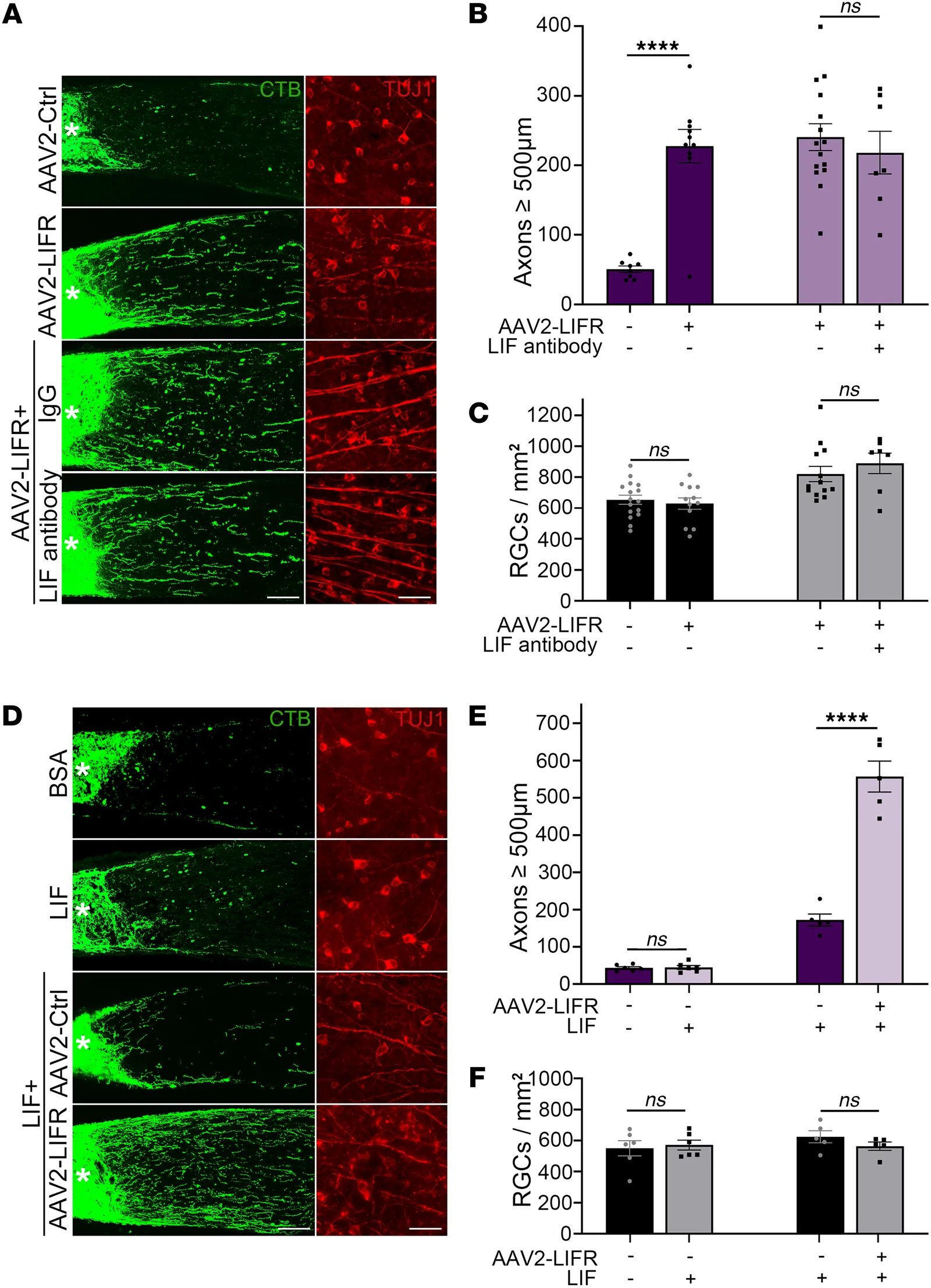
LIFR overexpression promotes axon regeneration in the mature optic nerve. (**A**) Regenerating axons and RGC survival in whole-mounted retinas visualized by fluorescent CTB (green) and immunostaining against βIII-tubulin (red), respectively, 2 weeks after ONC with the indicated treatments. An asterisk indicates the injury site. Scale bar: 100 μm (left), 50 μm (right). This experiment was conducted twice. (**B**) Quantification of regenerating axons. Statistical significance was determined by unpaired 2-tailed *t* test (n.s. *P* > 0.05, ****P < 0.0001; *n* = 7–15 nerves per group). (**C**) Quantification of RGC survival. Statistical significance was determined by unpaired 2-tailed *t* test (n.s. *P* > 0.05; *n* = 7–16 eyes per group). (**D**) Representative images for regenerating axons and RGC survival in whole-mounted retinas with indicated treatment. The asterisk indicates the injury site. Scale bar: 100 μm (left), 50 μm (right). This experiment was conducted once. (**E**) Quantification of regenerating axons. Statistical significance was determined by unpaired 2-tailed *t* test (n.s. *P* > 0.05, ****P < 0.0001; *n* = 5–6 nerves per group). (**F**) Quantification of RGC survival. Statistical significance was determined by unpaired 2-tailed *t* test (n.s. *P* > 0.05; *n* = 5–6 eyes per group). Data represent mean ± SEM.

**Figure 3:**
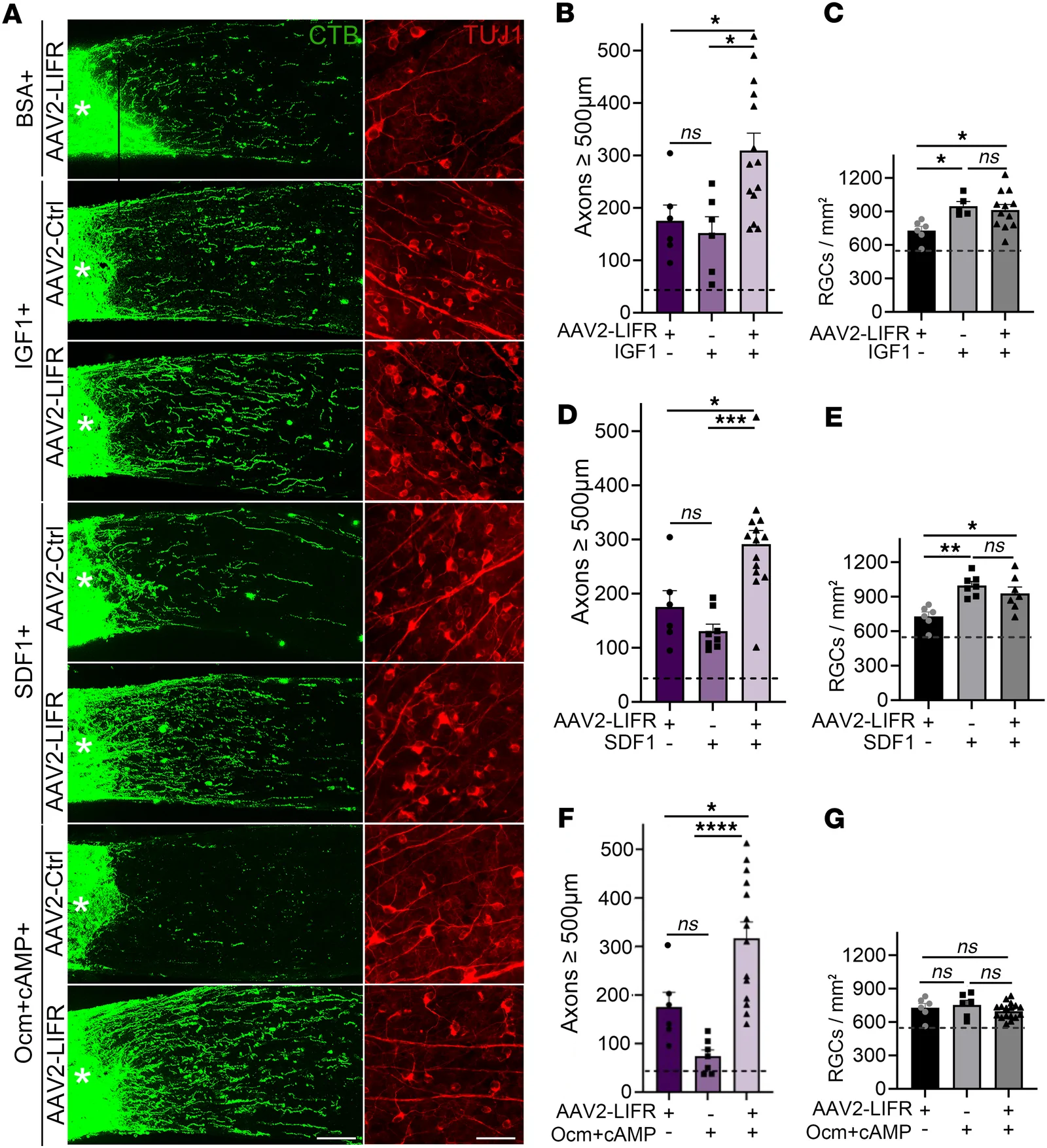
AAV2-LIFR enhances RGCs’ responsiveness to unrelated trophic factors. (**A**) Regenerating axons and RGC survival visualized by fluorescent CTB transport (green) and immunostaining against βIII-tubulin (red), respectively, 2 weeks after ONC with indicated treatments. Asterisk indicates injury site. Scale bar: 100 μm (left), 50 μm (right). This experiment was conducted once. (**B**, **D**, and **F**) Quantification of axon regeneration after IGF1 (**B**), SDF1 (**D**), and Ocm plus cAMP (**F**) treatment. Dashed lines in **B**, **D**, and **F** indicate regeneration in the BSA-treated control group. Statistical significance was determined by 1-way ANOVA followed by Tukey’s multiple-comparison test (n.s. *P* > 0.05, *P < 0.05, ***P < 0.001, ****P < 0.0001; *n* = 6–14 nerves per group). (**C**, **E**, and **G**) Quantification of RGC survival after IGF1 (**C**), SDF1 (**E**), and Ocm plus cAMP (**G**) treatment. Dashed lines in **C**, **E**, and **G** indicate survival in the BSA-treated control group. Statistical significance was determined by 1-way ANOVA followed by Tukey’s multiple-comparison test; n.s. *P* > 0.05, *P < 0.05, **P < 0.01; *n* = 5–12 eyes per group (**C**), 6–7 eyes per group (**E**), and 6–17 eyes per group (**G**). Data represent mean ± SEM.

**Figure 4:**
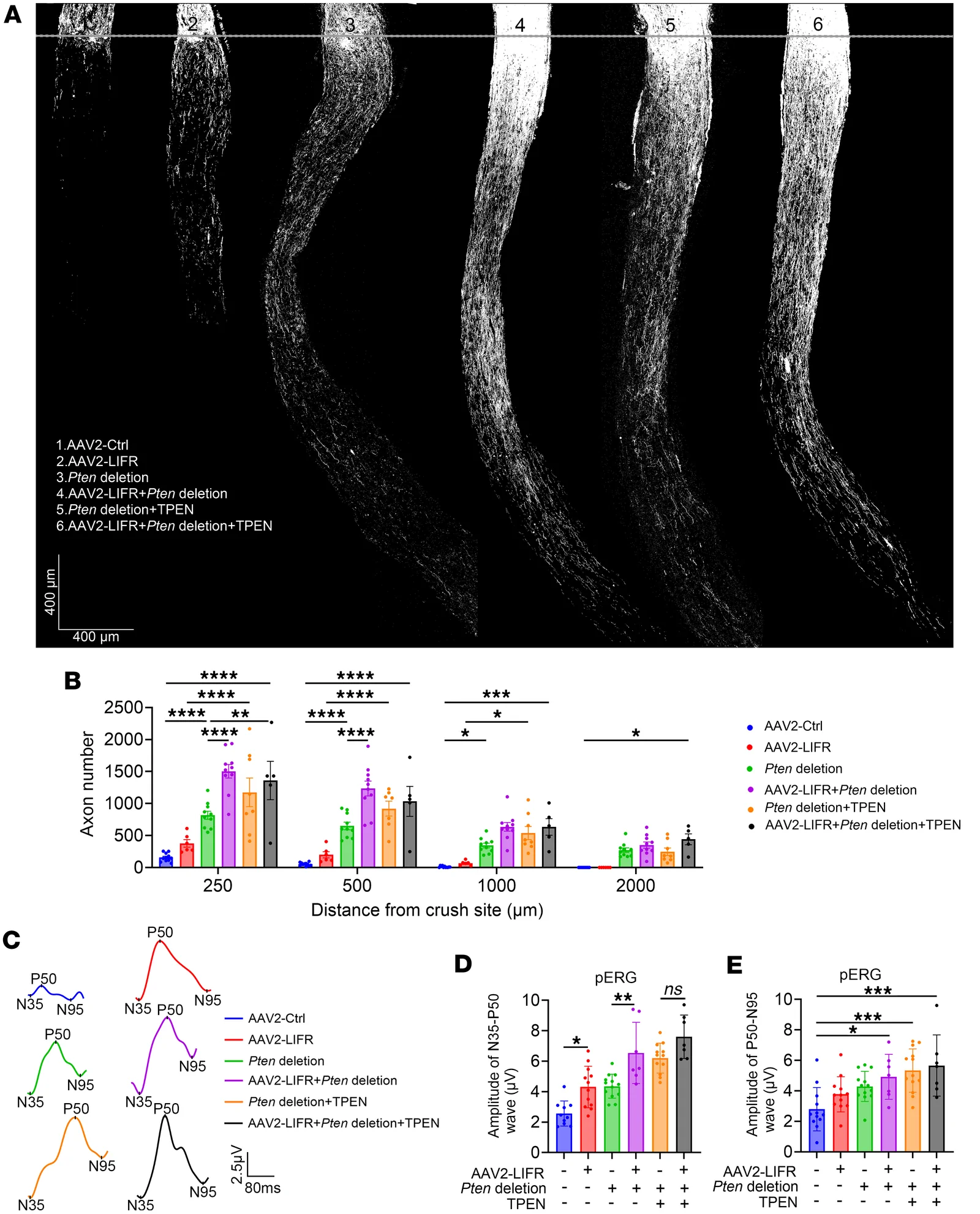
Long-term assessment of AAV2-LIFR–mediated optic nerve regeneration and visual function after optic nerve injury. (**A**) Representative images of axon regeneration visualized by CTB labeling (gray) at 8 weeks after ONC with the indicated treatments. The dashed line indicates the injury site. Scale bar: 400 μm. This experiment was conducted once. (**B**) Quantification of regenerating axons at 250, 500, 1,000, and 2,000 μm distal to the crush site with the indicated treatments. Statistical significance was determined by 2-way ANOVA followed by Tukey’s multiple-comparison test (*P < 0.05, **P < 0.01, ***P < 0.001, ****P < 0.0001; *n* = 5–11 nerves per group). (**C**) Representative pERG waveforms recorded at 8 weeks after ONC from each treatment cohort. N35, P50, and N95 peaks are labeled. Scale bars: vertical, 2.5 μV; horizontal, 80 ms. This experiment was conducted once. (**D** and **E**) Quantification of pERG N35-P50 (**D**) and P50-N95 (**E**) amplitudes. Statistical significance was determined by 1-way ANOVA followed by Tukey’s multiple-comparison test (n.s. *P* > 0.05, *P < 0.05, **P < 0.01, ***P < 0.001; *n* = 7–13 eyes per group). Data represent mean ± SEM.

**Figure 5:**
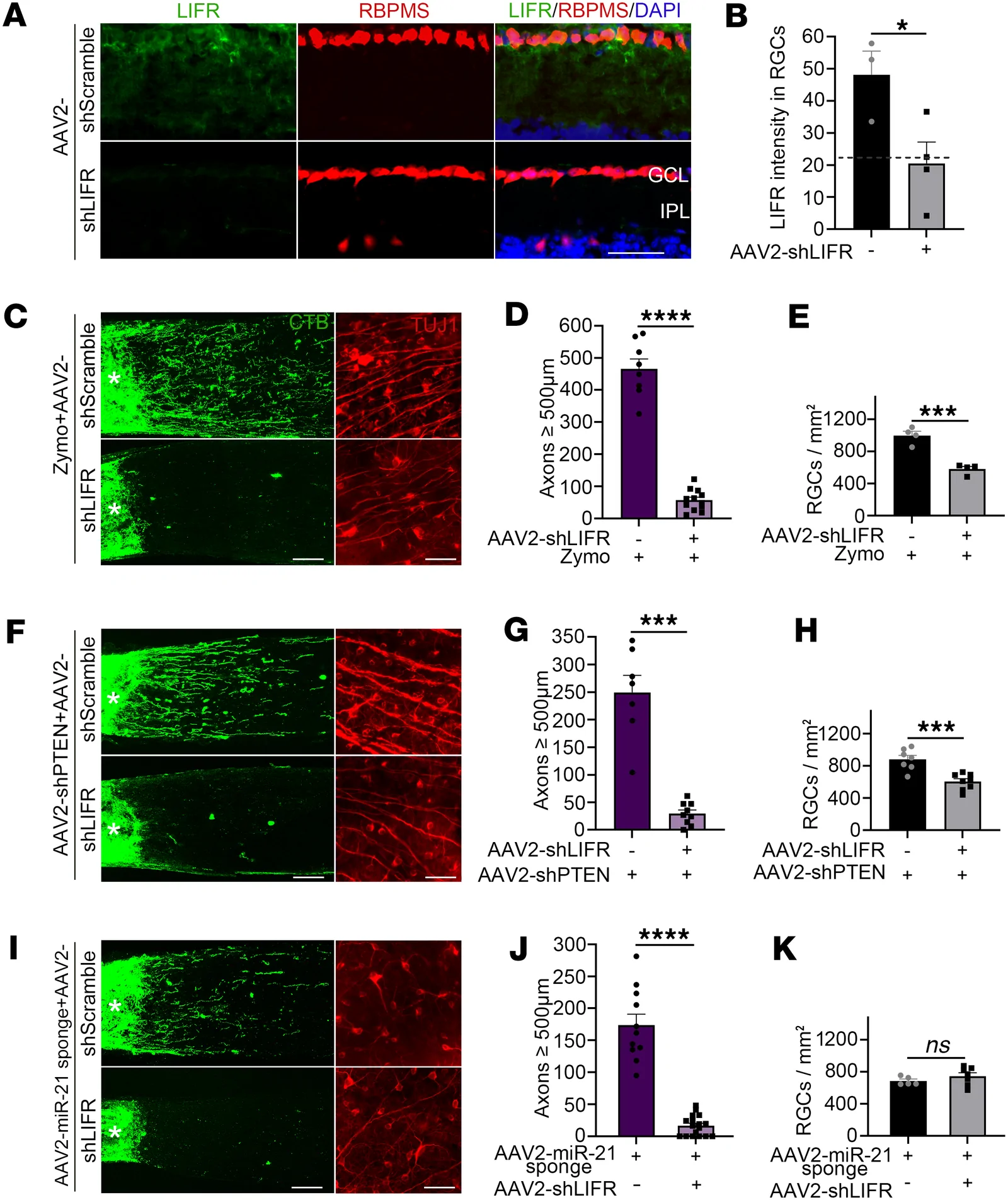
LIFR knockdown suppresses optic nerve regeneration. (**A**) Retinal cross sections from mice 2 weeks after AAV2-shScramble or AAV2-shLIFR injection immunostained for LIFR (green) and RBPMS (red) and stained with DAPI (blue). GCL, ganglion cell layer; IPL, inner plexiform layer. Scale bar: 30 μm. This experiment was conducted once. (**B**) Quantification of LIFR intensity in RGCs. Dashed line indicates LIFR intensity when the first antibody is omitted during immunostaining. Statistical significance was determined by unpaired 2-tailed *t* test (*P < 0.05; *n* = 3–4 eyes per group). (**C**, **F**, and **I**) Representative images of axon regeneration (green) and RGC survival (red) after LIFR knockdown combined with zymosan (**C**), AAV2-shPTEN (**F**), or AAV2-miR-21-sponge (**I**). An asterisk indicates the injury site. Scale bars: 100 μm (left) and 50 μm (right). The experiments in **C** and **F** were conducted once, and experiments in **I** were conducted twice. (**D**, **G**, and **J**) Quantification of axon regeneration after LIFR knockdown combined with zymosan (**D**), AAV2-shPTEN (**G**), or AAV2-miR-21-sponge (**J**). Statistical significance was determined by an unpaired 2-tailed *t* test; ***P < 0.001, ****P < 0.0001; *n* = 8–12 nerves (**D**), 7–9 nerves (**G**), and 11–17 nerves (**J**) per group. (**E**, **H**, and **K**) Quantification of RGC survival after LIFR knockdown combined with zymosan (**E**), AAV2-shPten (**H**), or AAV2-miR-21-sponge (**K**). Statistical significance was determined by an unpaired 2-tailed *t* test; n.s. *P* > 0.05, ***P < 0.001; *n* = 4 eyes (**E**), 7–8 eyes (**H**), and 5–7 eyes (**K**) per group. Data represent mean ± SEM.

**Figure 6:**
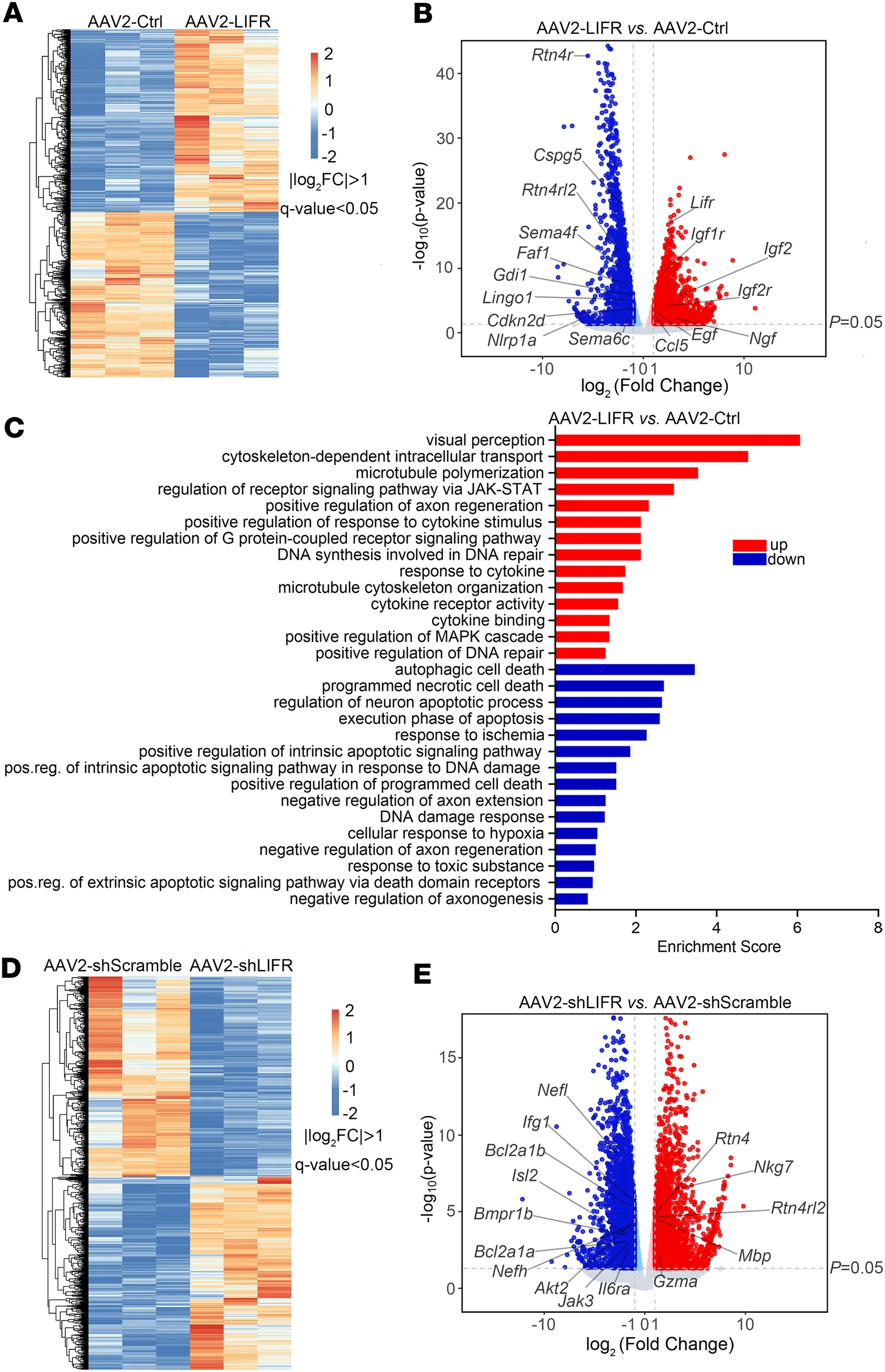
Transcriptional effects. (**A** and **D**) Heatmap showing the magnitude of upregulated (orange to red) and downregulated (blue) genes (*q* < 0.05, |log_2_FC| > 1) in RGCs 2 weeks after intraocular AAV2-LIFR (**A**, AAV2-LIFR vs. AAV2-Ctrl) or AAV2-shLIFR (**D**, AAV2-shLIFR vs. AAV2-shScramble, 3 replicates in each group). (**B** and **E**) Volcano plot showing individual upregulated (red) and downregulated (blue) genes in RGCs after transfection with AAV2-LIFR (**B**, AAV2-LIFR vs. AAV2-Ctrl) or AAV2-shLIFR (**E**, AAV2-shLIFR vs. AAV2-shScramble). (**C**) GO enrichment analysis of differentially expressed genes with indicated comparison in RGCs. Upregulated (red) and downregulated (blue) GO terms (AAV2-LIFR vs. AAV2-Ctrl).

**Figure 7:**
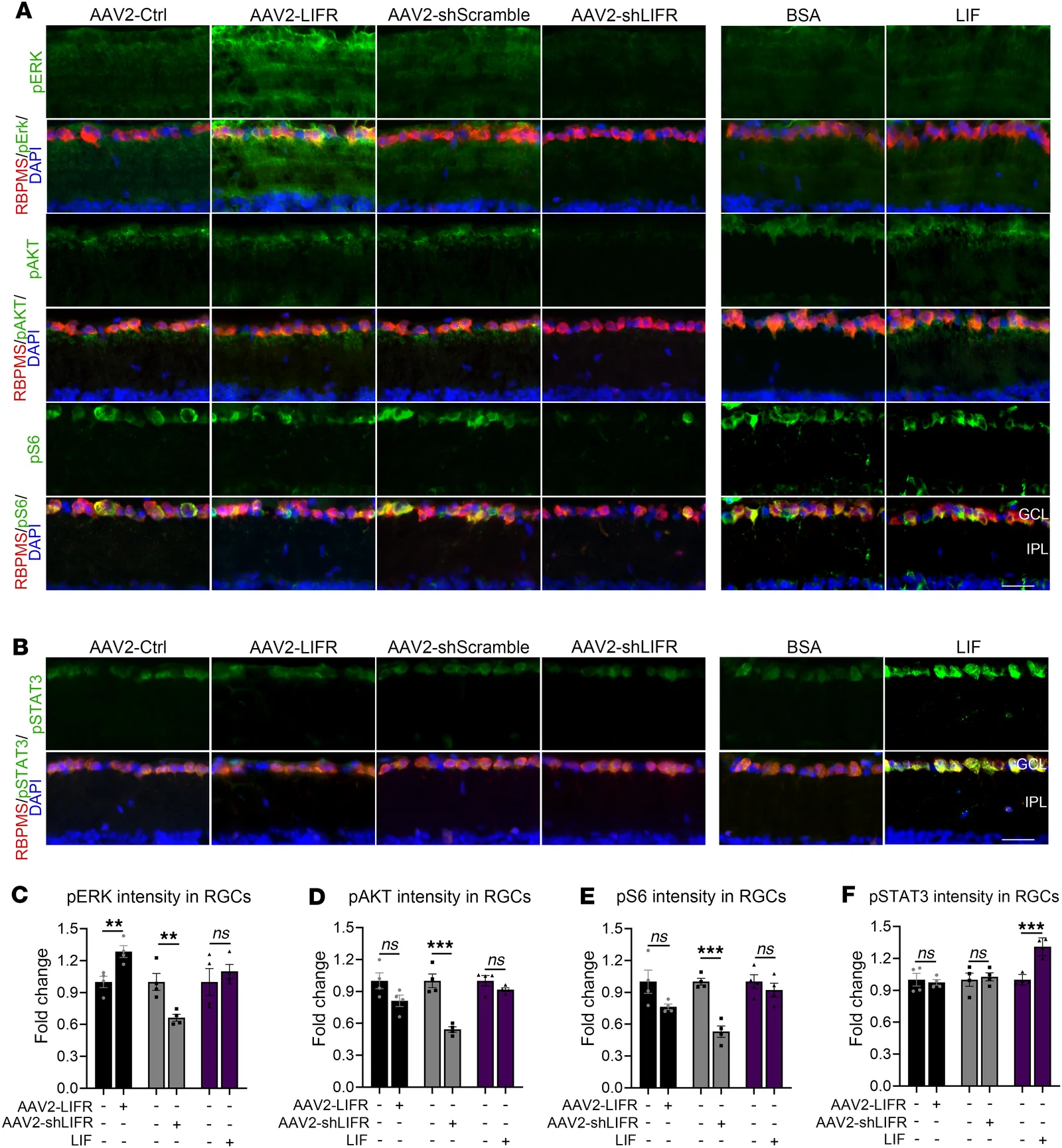
LIFR activates cell-signaling pathways related to CNS axon regeneration. (**A**) Retinal cross sections from mice 2 weeks after intraocular AAV2-Ctrl, AAV2-LIFR, AAV2-shScramble, AAV2-shLIFR, or 3 days after BSA or LIF injection, immunostained for pERK (green), pAKT (green), pS6 (green), RBPMS (red), and DAPI (blue). GCL, ganglion cell layer; IPL, inner plexiform layer. Scale bar: 30 μm. This experiment was conducted once. (**B**) Retinal cross sections immunostained for pSTAT3 (green), RBPMS (red), and DAPI (blue) after the same treatments as in **A**. Scale bar: 30 μm. This experiment was conducted once. (**C**–**F**) Quantification of pERK (**C**), pAKT (**D**), pS6 (**E**), and pSTAT3 (**F**) intensity in RBPMS^+^ RGCs with indicated treatments. Statistical significance was determined by unpaired 2-tailed *t* test (n.s. *P* > 0.05, **P < 0.01, ***P < 0.001; *n* = 4 per group). Data represent mean ± SEM.

**Figure 8:**
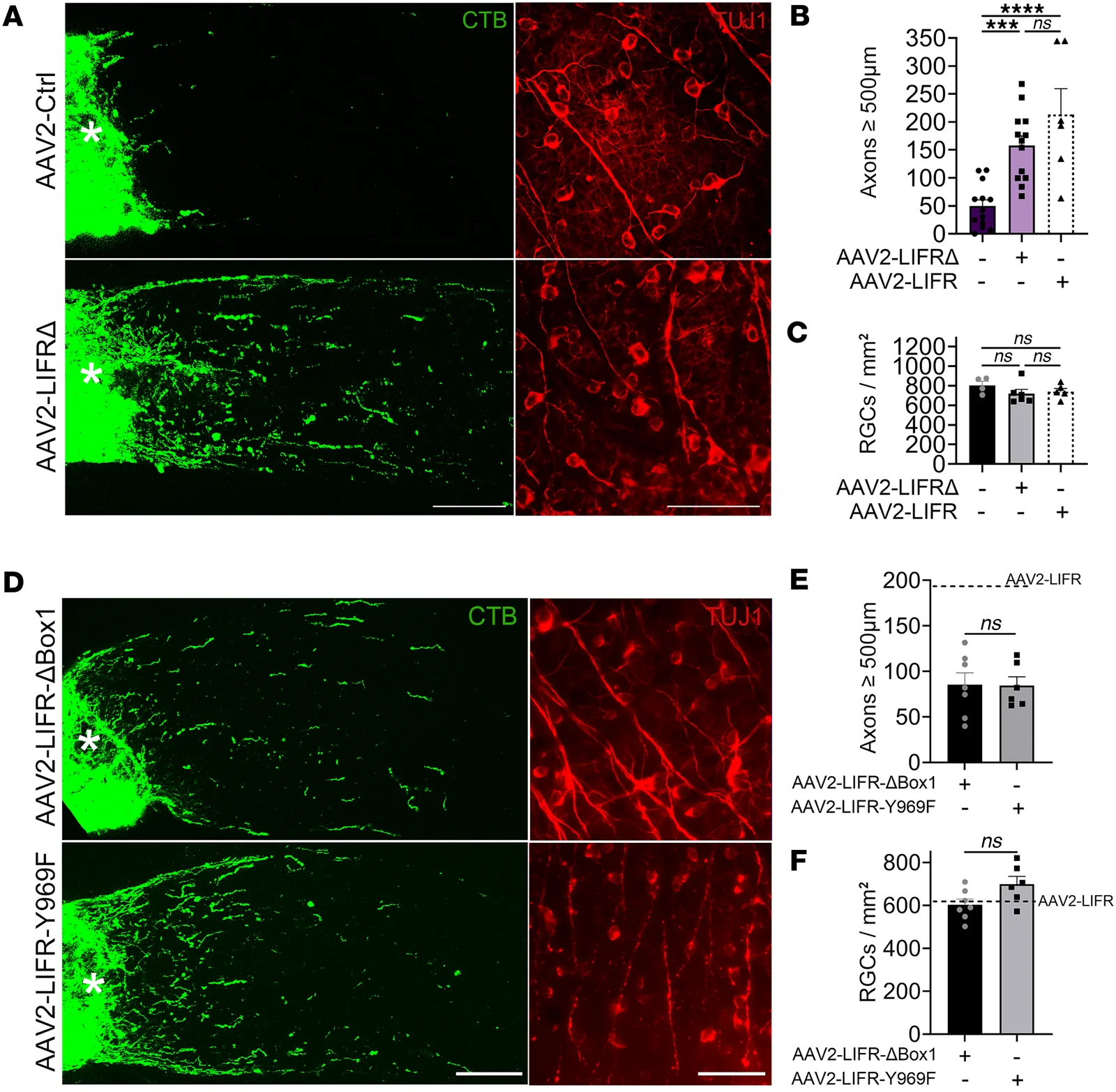
LIFR intracellular domain promotes regeneration. (**A**) Representative images of regenerating axons (CTB, green) and RGC survival (TUJ1, red) in mice treated with AAV2-LIFRΔ or AAV2-Ctrl. Asterisks indicate the injury site. Scale bars: 100 μm (left) and 75 μm (right). This experiment was conducted once. (**B** and **C**) Quantification of axon regeneration (**B**) and RGC survival (**C**) after AAV2-LIFRΔ treatment. Statistical significance was determined by 1-way ANOVA followed by Tukey’s multiple-comparison test; n.s. *P* > 0.05, ***P < 0.001, ****P < 0.0001; *n* = 6–13 nerves (**B**) and 4–6 retinas (**C**) per group. (**D**) Representative images of regenerating axons (CTB, green) and RGC survival (TUJ1, red) in mice treated with AAV2-LIFR-ΔBox1 or AAV2-LIFR-Y969F. Asterisk indicates the injury site. Scale bars: 100 μm (left) and 50 μm (right). This experiment was conducted once. (**E** and **F**) Quantification of axon regeneration (**E**) and RGC survival (**F**) after AAV2-LIFR-ΔBox1 or AAV2-LIFR-Y969F treatment. Dashed lines indicate the levels induced by AAV2-LIFR. Statistical significance was determined by unpaired 2-tailed *t* test; n.s. *P* > 0.05; *n* = 6–7 nerves (**E**) and 6–7 retinas (**F**) per group. Data represent mean ± SEM.

**Figure 9:**
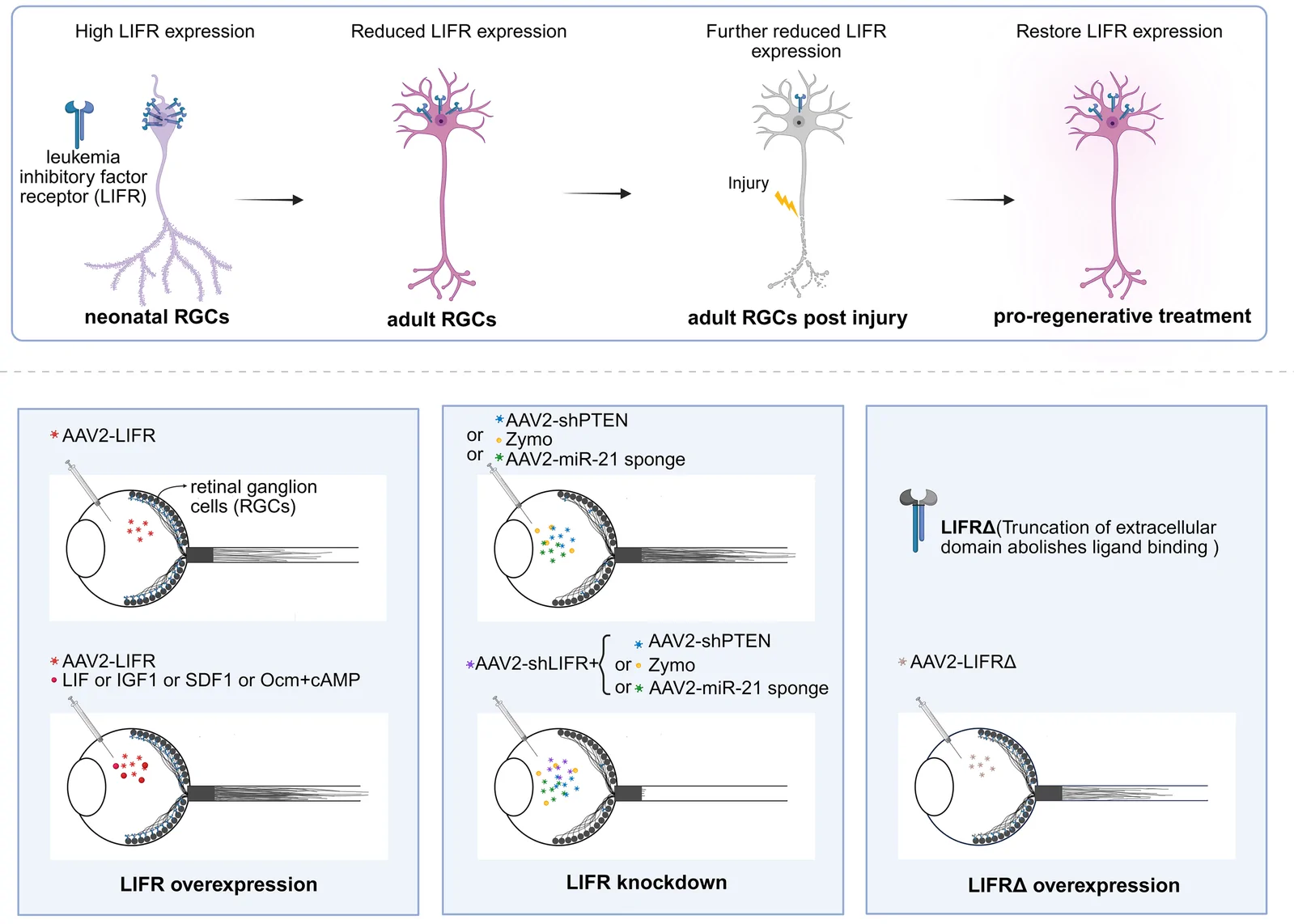
Schematic summary of the major findings. The expression of LIFR determines the regenerative potential and trophic responsiveness of RGCs. Compared with neonatal RGCs, LIFR is reduced in adult RGCs and is further reduced in adult RGCs after optic nerve injury. A pro-regenerative treatment (e.g., sterile intraocular inflammation) restores its expression to the normal mature level (top). LIFR overexpression in RGCs (intraocular AAV2-LIFR) promotes optic nerve regeneration independent of the ligand, LIF, and amplifies the effects of unrelated growth factors (e.g., IGF1, SDF1, Ocm: bottom left). In contrast, LIFR knockdown abolishes regeneration induced by potent stimuli such as PTEN knockdown, intraocular zymosan, or miR-21 inhibition with AAV2-miR-21-sponge (bottom center). A truncation of the extracellular domain of LIFR (LIFRΔ) also promotes regeneration (bottom right).

**Figure 10:**
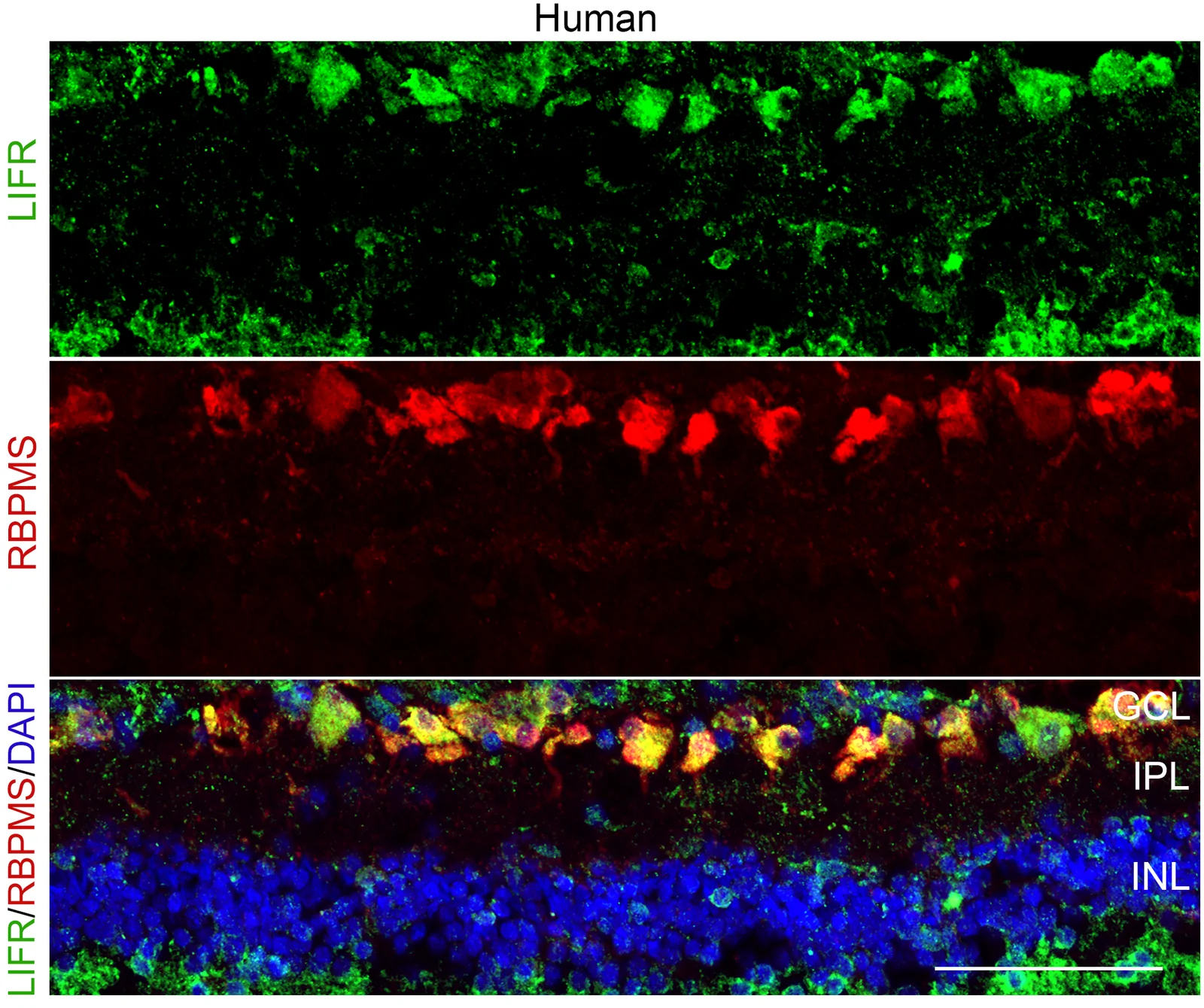
LIFR expression in human RGCs. Cross section from a human postmortem retina immunostained for LIFR (green), RBPMS (red), and DAPI (blue). GCL, ganglion cell layer; IPL, inner plexiform layer; INL, inner nuclear layer. Scale bar: 60 μm.

**Table 1:**
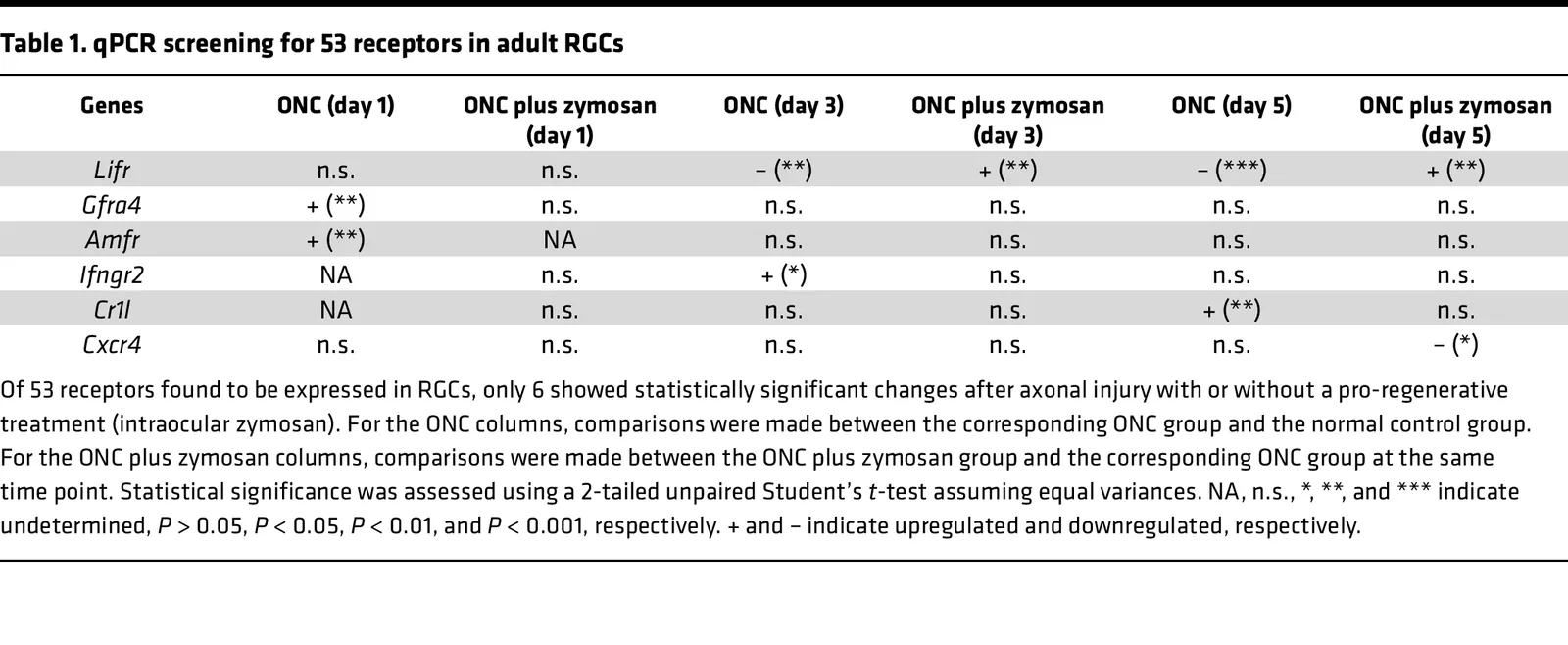
qPCR screening for 53 receptors in adult RGCs

## Version 1 09/01/2026

In-Press Preview

## Version 2 10/08/2026

Electronic publication
