# Supplementary material for: Optic nerve regeneration requires the intracellular domain of LIFRα/CD118
Source: JCI Insight. 2026 Sep 1;11(19):e202223. doi: 10.1172/jci.insight.202223 (PMC13726422; doi:10.1172/jci.insight.202223)
Supplement: Supplementary file 1 [file jciinsight-11-202223-s279.pdf]

Optic nerve regeneration requires the intracellular domain of LIFRα/CD118

Qian Jiang[1,2], Cong Wang[1,2], Yuerong Ren[1,2], Peiyun Duan[1,2], Ke Tian[1,2], Xiangwei Duan[1,2], Binghan Cai[1,2], Changzhong Xu[1,2], Ke Liu[1,2], Jian Li[3], Larry Benowitz[4], Ningli Wang[5], Bing Jiang[1,2], Lili Xie[1,2]**

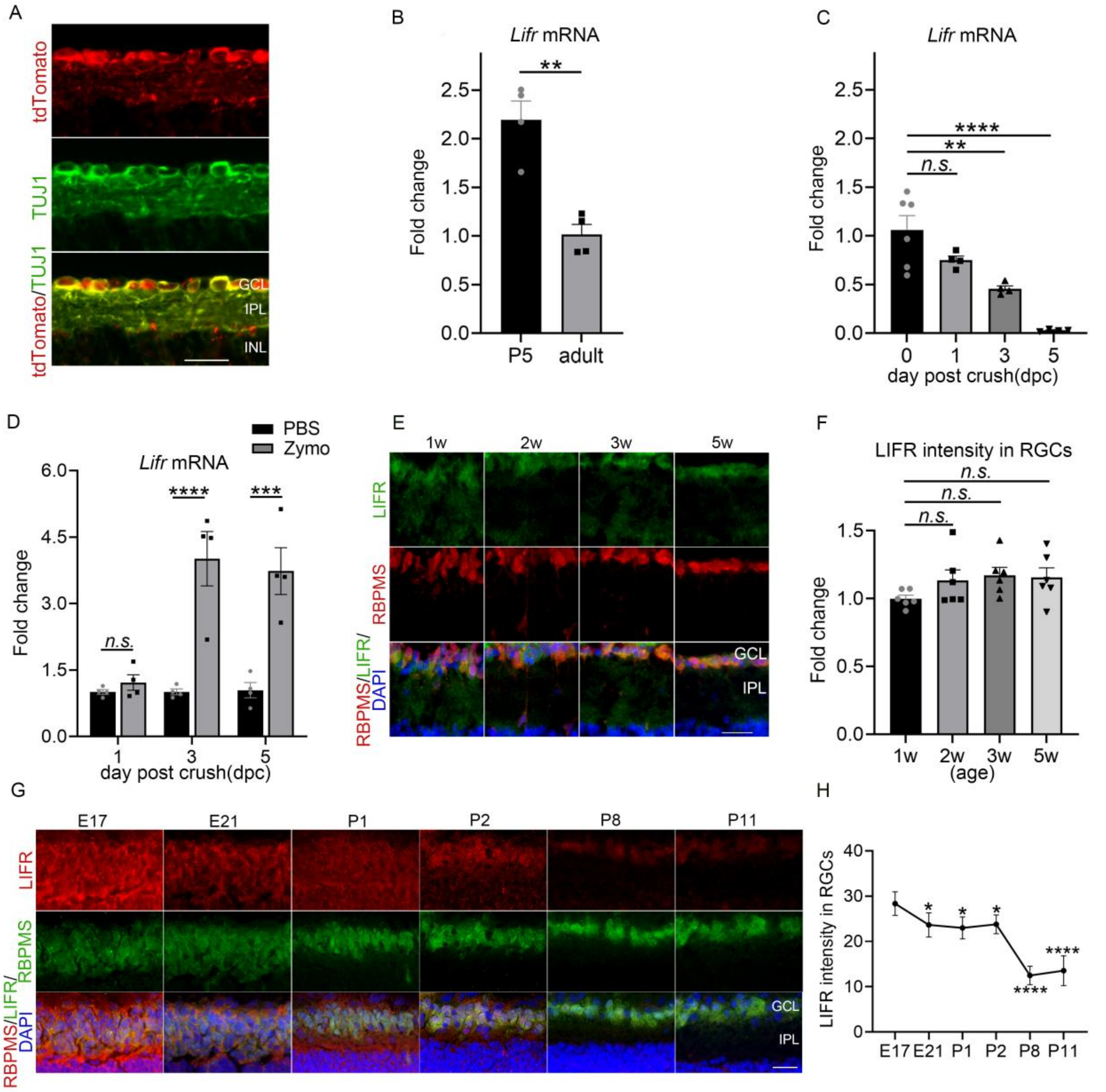

Supplemental Figure 1. LIFR intensity in RGCs during development.
(A) Retinal cross-sections from *Vglut2*-tdTomato mice showing genetic labeling of tdTomato (*red*) in TUJ1+ RGCs (*green*). Scale bar, 60µm. This experiment was conducted once.
(B) *Lifr* mRNA expression in RGCs from P5 and adult mice, normalized to adult controls. Statistical significance was determined by unpaired two-tailed t test. (** $P < 0.01$; n = 4 samples per group).
(C) *Lifr* mRNA expression in RGCs from adult mice at 1, 3, and 5 days post-crush (dpc), normalized to sham. Statistical significance was determined by one-way ANOVA followed by Tukey's multiple-comparison test (*n.s.* $P > 0.05$, ** $P < 0.01$, **** $P < 0.0001$; n = 4-6 samples per group).
(D) *Lifr* mRNA expression in adult RGCs at 1, 3, and 5 dpc with intraocular PBS or zymosan, normalized to time-matched PBS controls. Statistical significance was determined by two-way ANOVA followed by Šídák's multiple-comparison test (*n.s.*: $P > 0.05$, *** $P < 0.001$, **** P < 0.0001; n = 4 samples per group).
(E) Retinal cross-sections from mice aged 1w, 2w, 3w and 5w immunostained for LIFR (*green*), RBPMS (*red*) and DAPI (*blue*). GCL: ganglion cell layer, IPL: inner plexiform layer. Scale bar, 30 µm. This experiment was conducted once.
(F) Quantification of LIFR intensity in RGCs in (E), normalized to the 1-week group. Statistical significance was determined by one-way ANOVA followed by Tukey's multiple-comparison test (*n.s.*: $P > 0.05$; n=6 eyes per group).
(G) Retinal cross-sections from rats at embryonic day 17 (E17), E21, P1, P2, P8 and P11 immunostained with LIFR (*red*), RBPMS (*green*) and DAPI (*blue*). GCL: ganglion cell layer, IPL: inner plexiform layer. Scale bar, 30 µm. This experiment was conducted once.
(H) Quantification of LIFR intensity in RGCs in **(G)**. Statistical significance was determined by one-way ANOVA followed by Tukey's multiple-comparison test. Statistical comparisons are relative to the E17 group (* $P < 0.05$, **** $P < 0.0001$; n = 6 eyes per group).
Data represent mean ± SEM.

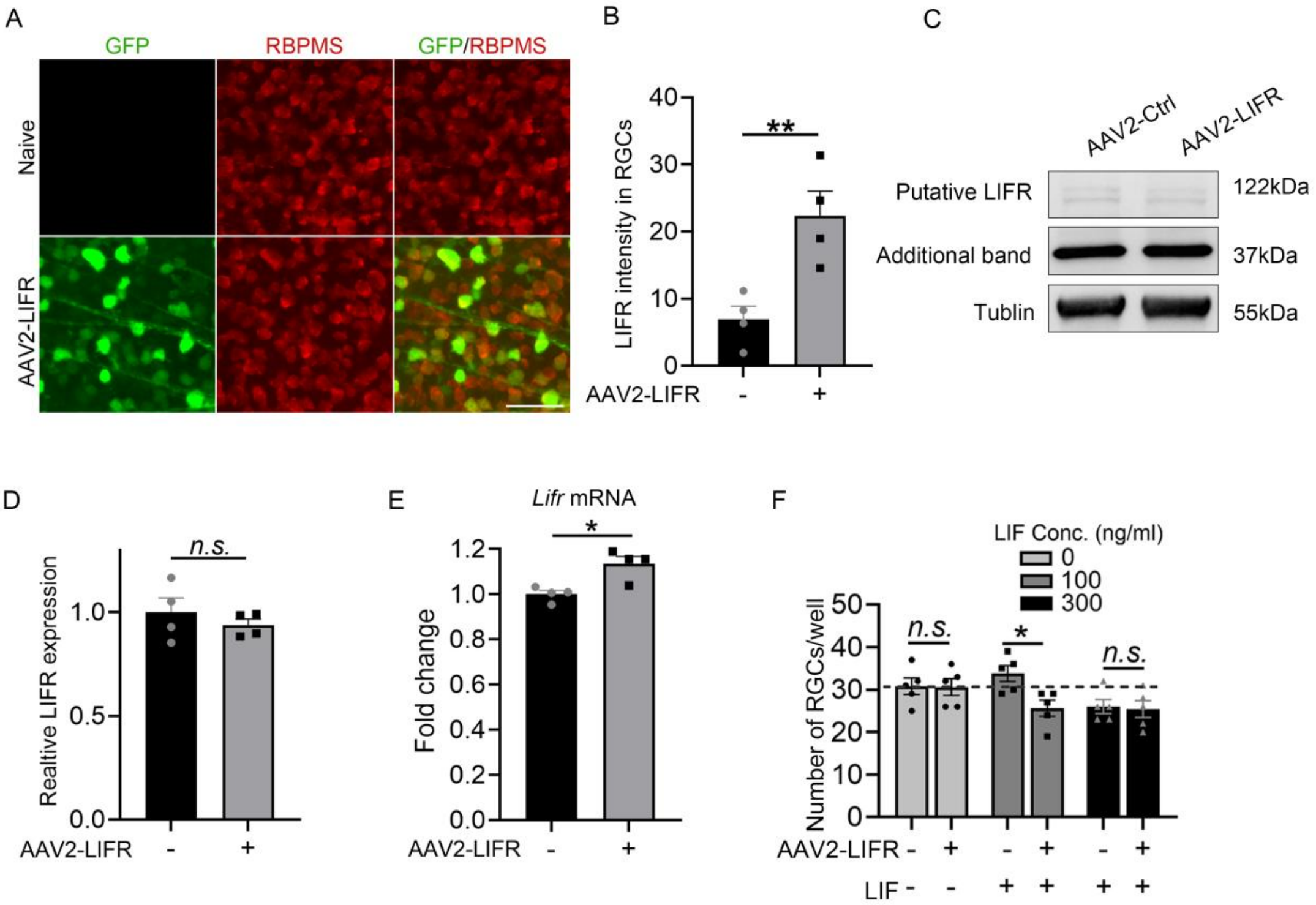

Supplemental Figure 2. LIFR overexpression does not increase adult RGC survival.

(A) Representative retinal flat-mount images showing GFP expression and RBPMS-positive RGCs in naïve and AAV2-LIFR-injected retinas. Scale bar, 50 µm. This experiment was conducted once.

(B) Quantification of LIFR intensity in adult mice two weeks following intraocular AAV2-LIFR. Statistical significance was determined by unpaired two-tailed t test. (** *P*< 0.01; n=4 eyes per group).

(C) Representative whole-retina immunoblots for putative LIFR, an additional band, and tubulin two weeks after AAV2-Ctrl or AAV2-LIFR injection. This experiment was conducted once.

(D) Quantification of putative LIFR protein levels in whole-retina lysates. Statistical significance was determined by unpaired two-tailed t test (*n.s.*: $P > 0.05$; n =4 retinas per group).

(E) Whole-retina qPCR analysis of *Lifr* mRNA expression two weeks after AAV2-Ctrl or AAV2-LIFR injection. Statistical significance was determined by unpaired two-tailed t test (*$P < 0.05$; n = 4 retinas per group).

(F) Quantification of RGC survival from *Vglut2*-tdTomato mice with indicated treatments after culturing for 3 days. Statistical significance was determined by unpaired two-tailed t test (*n.s.:* $P > 0.05$, *$P < 0.05$; n = 5 wells per group).

Data represent mean ± SEM.

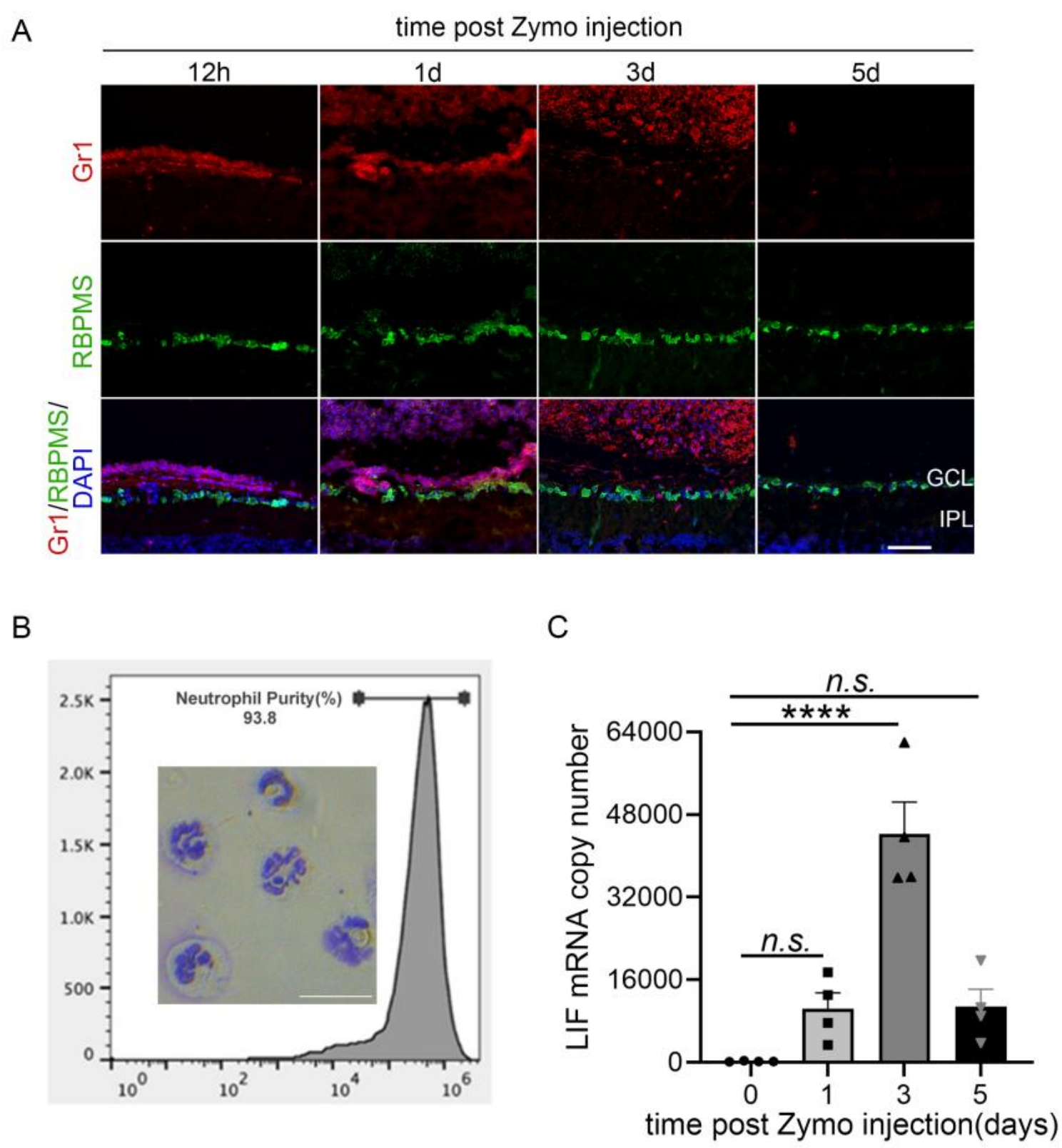

Supplemental Figure 3. Infiltrating neutrophils express Lif after intraocular zymosan.

(A) Retinal cross-sections from mice at different time points following intraocular zymosan immunostained with Gr1 (*red*), RBPMS (*green*) and DAPI (*blue*) to visualize neutrophil infiltration. GCL: ganglion cell layer, IPL: inner plexiform layer. Scale bar, 30 µm. This experiment was conducted once.

(B) Detection of the purification of MACS-sorted neutrophils by flow cytometry (n=3 replicates). Giemsa staining indicated the typical multilobed nuclear morphology of isolated neutrophils. Scale bar, 20 µm in inset panel.

(C) *Lif* mRNA level in isolated neutrophils at 1, 3, and 5d post introduction of zymosan. Statistical significance was determined by one-way ANOVA followed by Tukey's multiple-comparison test (*n.s.* $P > 0.05$, **** $P < 0.0001$; n = 4 samples per group).

Data represent mean ± SEM.

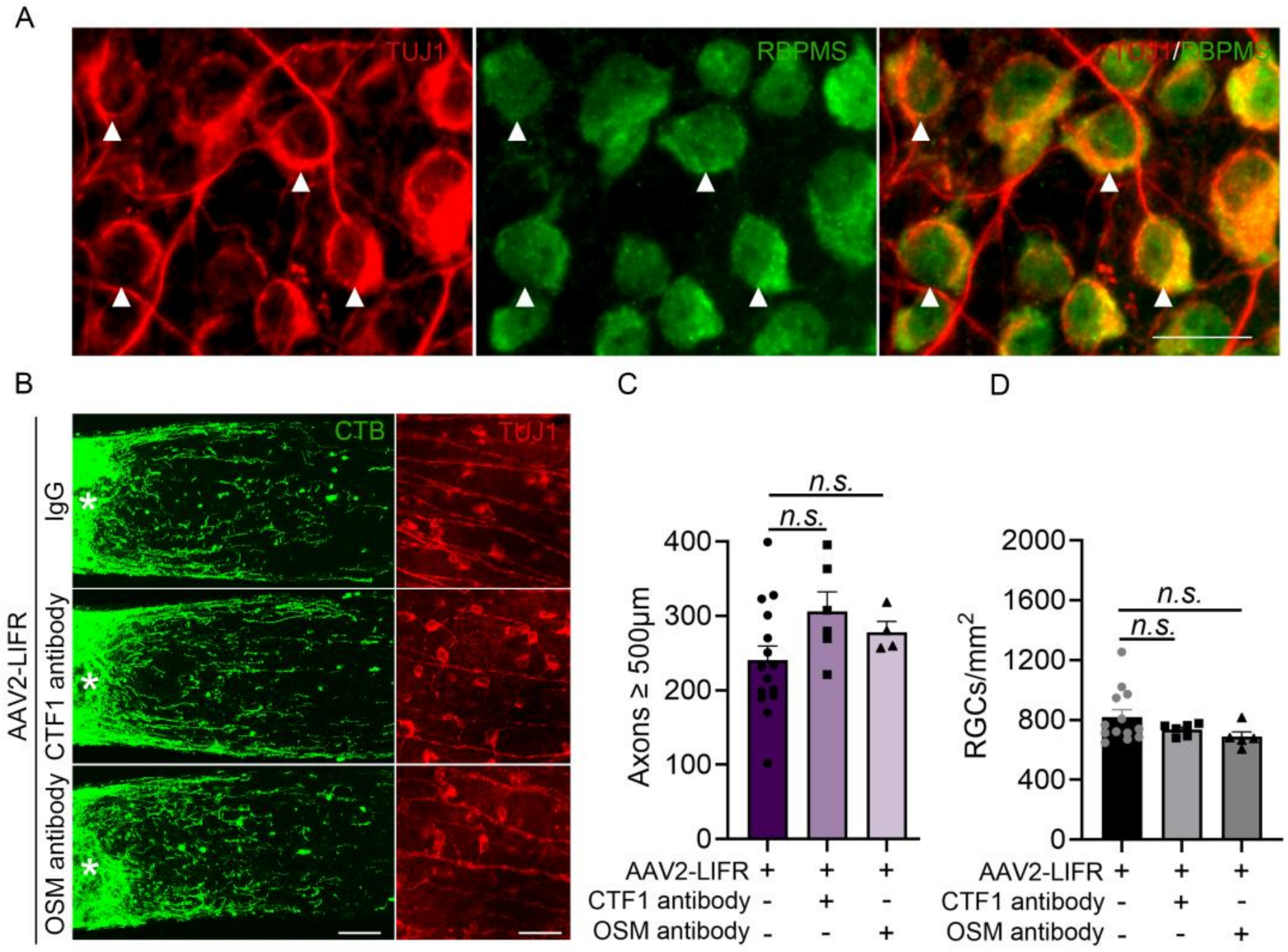

Supplemental Figure 4. The effect of LIFR overexpression is ligand-independent.

(A) Intact whole-mounted retinas immunostained for TUJ1 (*red*) and RBPMS (*green*). TUJ1 staining was only seen in RBPMS-positive RGCs. Scale bar, 10 µm. This experiment was conducted once.

(B) Regenerating axons and RGC survival visualized by fluorescent CTB (*green*) and immunostaining against βIII-tubulin (*red*), respectively after intraocular AAV2-LIFR combined with CTF1 neutralizing antibody, OSM neutralizing antibody, or the isotype control. Scale bar, 100 µm (left) , 50 µm (right) panel. This experiment was conducted once.

(C) Quantification of regenerating axons. Statistical significance was determined by one-way ANOVA followed by Tukey's multiple-comparison test (*n.s.* $P > 0.05$; n = 4-15 nerves per group).

(D) Quantification of RGC survival. Statistical significance was determined by one-way ANOVA followed by Tukey's multiple-comparison test (*n.s.* $P > 0.05$; n = 5-13 eyes per group).

Data represent mean ± SEM.

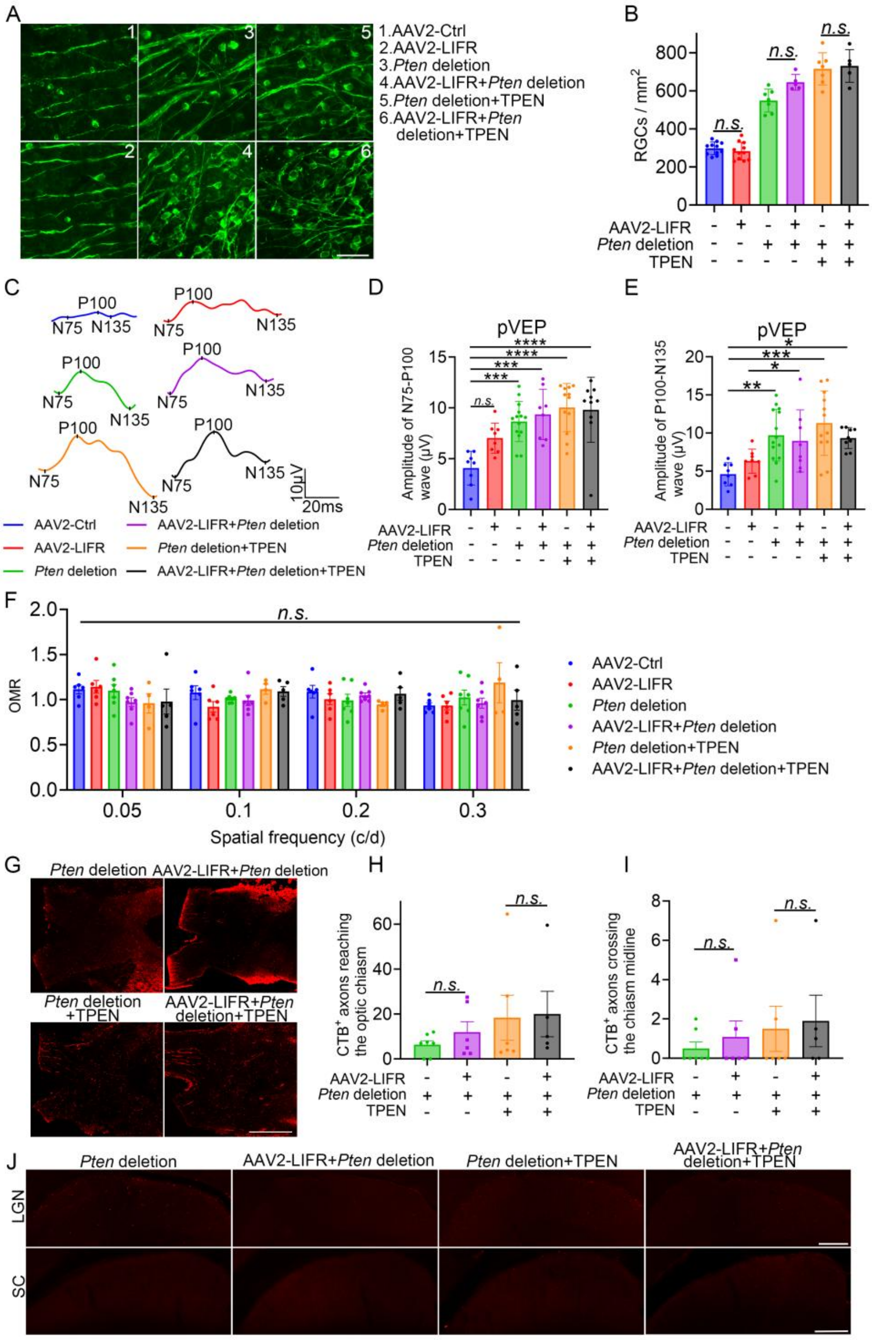
A
1.AAV2-Ctrl
2.AAV2-LIFR
3.Pten deletion
4.AAV2-LIFR+Pten deletion
5.Pten deletion+TPEN
6.AAV2-LIFR+Pten deletion+TPEN
B
RGCs / mm²
n.s.
AAV2-LIFR
Pten deletion
TPEN
C
P100
N75
N135
10μV
20ms
AAV2-Ctrl
AAV2-LIFR
Pten deletion
AAV2-LIFR+Pten deletion
Pten deletion+TPEN
AAV2-LIFR+Pten deletion+TPEN
D
pVEP
Amplitude of N75-P100 wave (μV)
E
pVEP
Amplitude of P100-N135 wave (μV)
F
OMR
Spatial frequency (c/d)
G
Pten deletion
AAV2-LIFR+Pten deletion
Pten deletion +TPEN
AAV2-LIFR+Pten deletion+TPEN
H
CTB⁺ axons reaching the optic chiasm
I
CTB⁺ axons crossing the chiasm midline
J
Pten deletion
AAV2-LIFR+Pten deletion
Pten deletion+TPEN
AAV2-LIFR+Pten deletion+TPEN
LGN
SC

Supplemental Figure 5. Long-term assessment of RGC survival, central target innervation, and visual function 8 weeks after optic nerve injury.

(A) RGC survival in whole-mounted retinas visualized by immunostaining against βIII-tubulin (TUJ1, *green*) 8 weeks after ONC with indicated treatments. Scale bar, 100μm. This experiment was conducted once.

(B) Quantification of RGC survival. Statistical significance was determined by one-way ANOVA followed by Tukey's multiple-comparison test. (*n.s.* $P > 0.05$; n = 5-11 eyes per group).

(C) Representative pVEP waveforms recorded at 8 weeks after ONC from the indicated treatment groups. N75, P100 and N135 peaks are indicated. Scale bars: vertical= 10 μV, horizontal=20 ms. This experiment was conducted once.

(D and E) Quantification of pVEP N75-P100 **(D)** and P100-N135 **(E)** amplitudes. Statistical significance was determined by one-way ANOVA followed by Tukey's multiple-comparison test. (*n.s.*: $P > 0.05$, * $P < 0.05$, ** $P < 0.01$, *** $P < 0.001$, **** $P < 0.0001$; n = 8-14 eyes per group).

(F) Quantification of optomotor responses at spatial frequencies of 0.05, 0.1, 0.2, and 0.3 cycles/degree at 8 weeks after ONC. Statistical significance was determined by two-way ANOVA followed by Tukey's multiple-comparison test. (*n.s.*: $P > 0.05$; n = 4-7 mice per group).

(G) Representative CTB-labeled axon images at the optic chiasm 8 weeks after ONC for the indicated treatment groups. Scale bar, 400 μm. This experiment was conducted once.

(H and I) Quantification of CTB-positive axons reaching the optic chiasm (H) and crossing the chiasm midline (I). Statistical significance was determined by one-way ANOVA followed by Tukey's multiple-comparison test. (*n.s.*: $P > 0.05$; n = 5-7 mice per group).

(J) Representative images of CTB-labeled axons in the LGN (upper panels) and SC (lower panels) at 8 weeks after ONC in the indicated groups. Scale bars: both 200μm. This experiment was conducted once.

Data represent mean ± SEM.

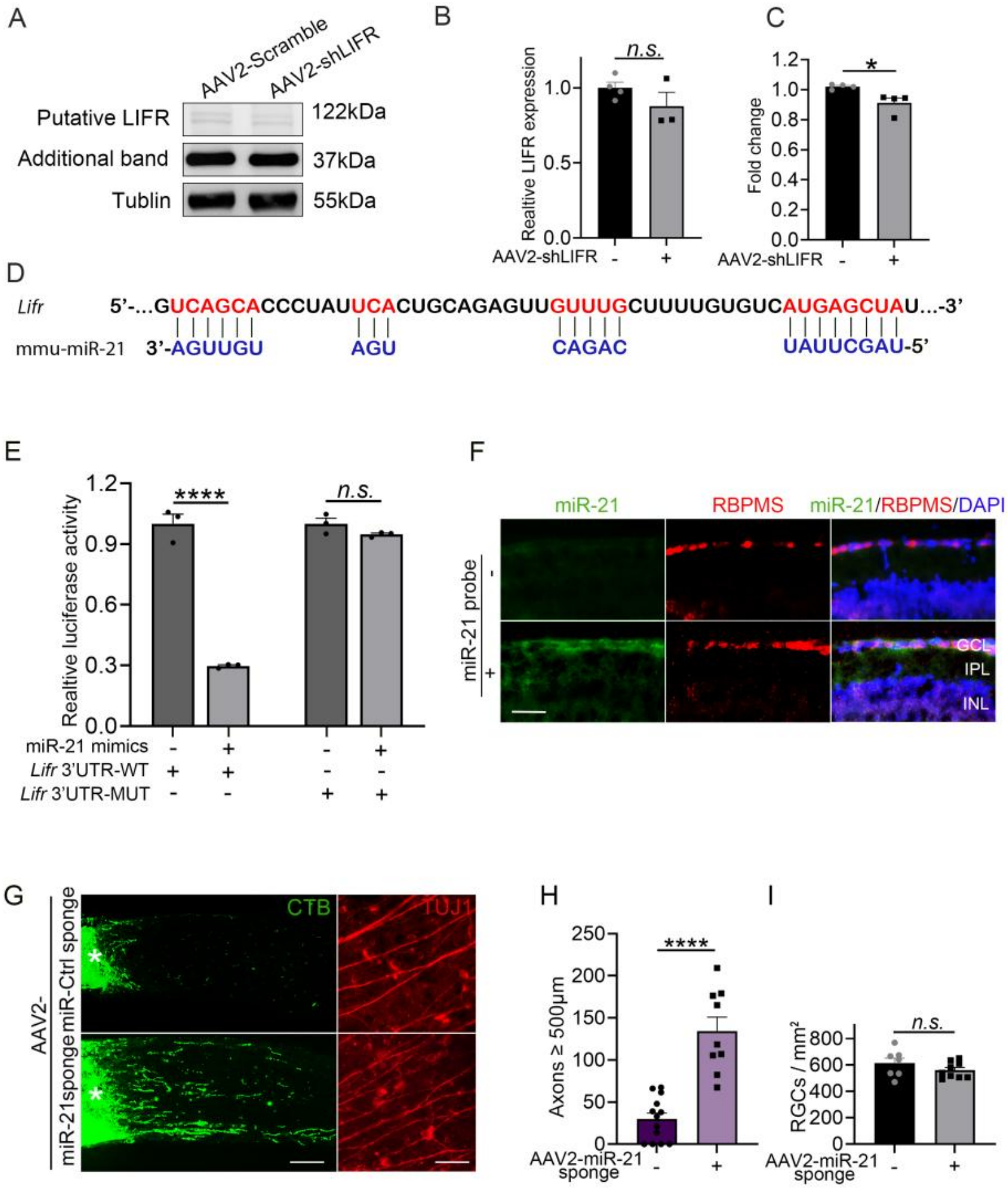
A
AAV2-Scramble
AAV2-shLIFR
Putative LIFR
Additional band
Tublin
122kDa
37kDa
55kDa
B
n.s.
Realtive LIFR expression
AAV2-shLIFR
C
*
Fold change
AAV2-shLIFR
D
Lifr
5'-...GUCAGCACCCUAUUCACUGCAGAGUUGUUUGCUUUUGUGUCAUGAGCUAU...-3'
mmu-miR-21
3'-AGUUGU
AGU
CAGAC
UAUUCGAU-5'
E
****
n.s.
Realtive luciferase activity
miR-21 mimics
Lifr 3'UTR-WT
Lifr 3'UTR-MUT
F
miR-21
RBPMS
miR-21/RBPMS/DAPI
miR-21 probe
GCL
IPL
INL
G
CTB
TUJ1
AAV2-
miR-Ctrl sponge
miR-21sponge
H
****
Axons ≥ 500μm
AAV2-miR-21 sponge
I
n.s.
RGCs / mm²
AAV2-miR-21 sponge

Supplemental Figure 6. Validation of LIFR knockdown and AAV2-miR-21-sponge promotes axon regeneration

(A) Representative whole-retina immunoblots for putative LIFR, an additional band, and Tubulin two weeks after AAV2-shScramble or AAV2-shLIFR injection. This experiment was conducted once.

(B) Quantification of putative LIFR protein levels in whole-retina lysates. Statistical significance was determined by unpaired two-tailed t test (*n.s.*: $P > 0.05$; n =3-4 retinas per group).

(C) Whole-retina qPCR analysis of *Lifr* mRNA expression two weeks after AAV2-shScramble or AAV2-shLIFR injection. Statistical significance was determined by unpaired two-tailed t test (* $P < 0.05$; n =4 retinas per group).

(D) Colored sequences indicate the binding site between mouse miR-21 and the 3'-UTR of *Lifr* mRNA.

(E) Dual-luciferase reporter assay demonstrating the direct interaction between miR-21 and the 3'-UTR of *Lifr* mRNA. Statistical significance was determined by one-way ANOVA followed by Tukey's multiple-comparison test (*n.s.*: $P > 0.05$, **** $P < 0.0001$; n=3 wells per group).

(F) Fluorescence in situ hybridization (FISH) showing high expression of miR-21 (*green*) in RBPMS+ (*red*) RGCs. This experiment was conducted once.

(G) Regenerating axons and RGC survival visualized by fluorescent CTB (*green*) and immunostaining against βIII-tubulin (*red*) respectively two weeks after ONC with indicated treatments. The asterisk indicates the injury site. Scale bar, 100 µm (left), 50 µm (right) . This experiment was conducted once.

(H and I) Quantification of regenerating axons (H) and RGC survival (I) after AAV2-miR-21 sponge treatment. Statistical significance was determined by unpaired two-tailed t test (AAV2-miR-21 sponge *vs.* AAV2-Ctrl sponge, *n.s.*: $P > 0.05$, **** $P < 0.0001$; n = 9-13 nerves (H) and 7-9 eyes (I) per group).

Data represent mean ± SEM.

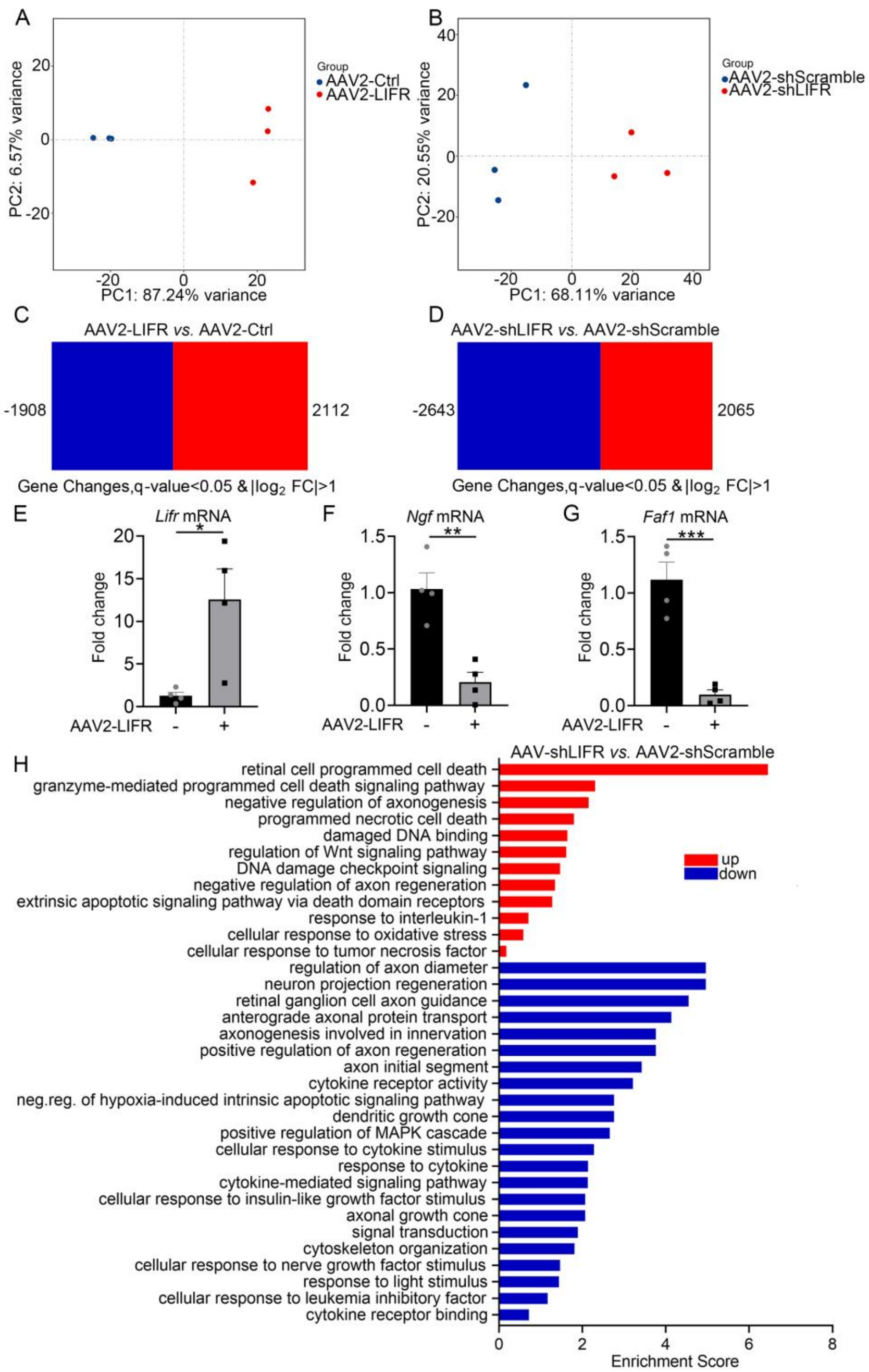
A
PC2: 6.57% variance
PC1: 87.24% variance
Group
AAV2-Ctrl
AAV2-LIFR
B
PC2: 20.55% variance
PC1: 68.11% variance
Group
AAV2-shScramble
AAV2-shLIFR
C
AAV2-LIFR vs. AAV2-Ctrl
-1908
2112
Gene Changes,q-value<0.05 & |log2 FC|>1
D
AAV2-shLIFR vs. AAV2-shScramble
-2643
2065
Gene Changes,q-value<0.05 & |log2 FC|>1
E
Lifr mRNA
Fold change
AAV2-LIFR
F
Ngf mRNA
Fold change
AAV2-LIFR
G
Faf1 mRNA
Fold change
AAV2-LIFR
H
AAV-shLIFR vs. AAV2-shScramble
retinal cell programmed cell death
granzyme-mediated programmed cell death signaling pathway
negative regulation of axonogenesis
programmed necrotic cell death
damaged DNA binding
regulation of Wnt signaling pathway
DNA damage checkpoint signaling
negative regulation of axon regeneration
extrinsic apoptotic signaling pathway via death domain receptors
response to interleukin-1
cellular response to oxidative stress
cellular response to tumor necrosis factor
regulation of axon diameter
neuron projection regeneration
retinal ganglion cell axon guidance
anterograde axonal protein transport
axonogenesis involved in innervation
positive regulation of axon regeneration
axon initial segment
cytokine receptor activity
neg.reg. of hypoxia-induced intrinsic apoptotic signaling pathway
dendritic growth cone
positive regulation of MAPK cascade
cellular response to cytokine stimulus
response to cytokine
cytokine-mediated signaling pathway
cellular response to insulin-like growth factor stimulus
axonal growth cone
signal transduction
cytoskeleton organization
cellular response to nerve growth factor stimulus
response to light stimulus
cellular response to leukemia inhibitory factor
cytokine receptor binding
up
down
Enrichment Score

Supplemental Figure 7. PCA, differential gene expression analysis, and qPCR validation of LIFR-regulated genes in adult RGCs.

(A and B) Principal component analysis (PCA) showing the variance in gene expression profiles in adult RGCs following transfection of AAV2-LIFR (A, AAV2-LIFR *vs.* AAV2-Ctrl) or AAV2-shLIFR (B, AAV2-shLIFR *vs.* AAV2-shScramble).

(C and D) Box plots showing the number of DEGs ($q < 0.05$, $|\log_2 FC| > 1$) in RGCs two weeks following intraocular AAV2-LIFR (C, AAV2-LIFR *vs.* AAV2-Ctrl) or AAV2-shLIFR (D, AAV2-shLIFR *vs.* AAV2-shScramble).

(E-G) qPCR validation of selected LIFR-regulated genes in FACS-isolated adult RGCs after AAV2-Ctrl or AAV2-LIFR injection: *Lifr* (E), *Ngf* (F) and *Faf1* (G). Statistical significance was determined by unpaired two-tailed t test (* $P < 0.05$, ** $P < 0.01$, *** $P < 0.001$; n = 4 replicates per group).

(H) GO enrichment analysis of differentially expressed genes with indicated comparison in RGCs. Up-regulated (*red*) and down-regulated (blue) GO terms following AAV2-shLIFR transfection (n = 3 replicates per group, AAV2-shLIFR *vs*. AAV2-shScramble).

Data represent mean ± SEM.

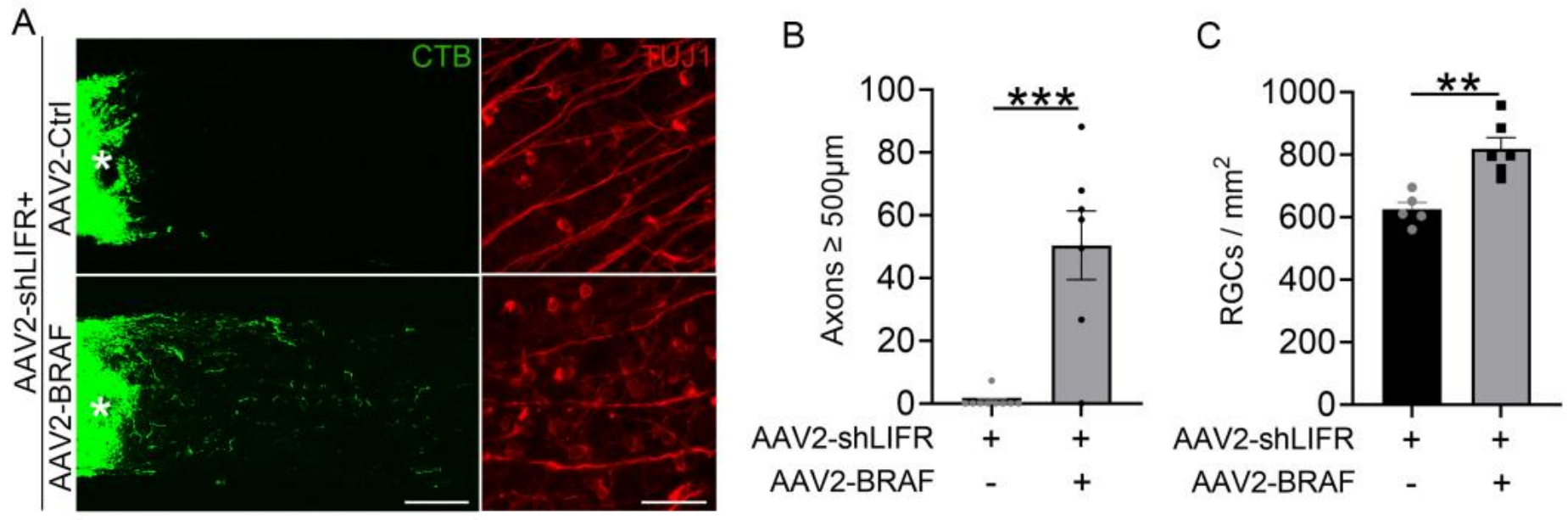

Supplemental Figure 8. BRAF partially restores axon regeneration and RGC survival following LIFR knockdown.

(A) Representative images of regenerating axons (CTB, green) and surviving RGCs (TUJ1, red) in AAV2-shLIFR-treated mice with or without AAV2-BRAF. Asterisk indicates the injury site. Scale bars: 100 µm (left) and 50 µm (right). This experiment was conducted once.

(B and C) Quantification of axon regeneration (B) and RGC survival (C). Statistical significance was determined by unpaired two-tailed t test. (***P* < 0.01, ****P* < 0.001; n=7-9 nerves (B) and 5-6 retinas (C) per group).

Data represent mean ± SEM.

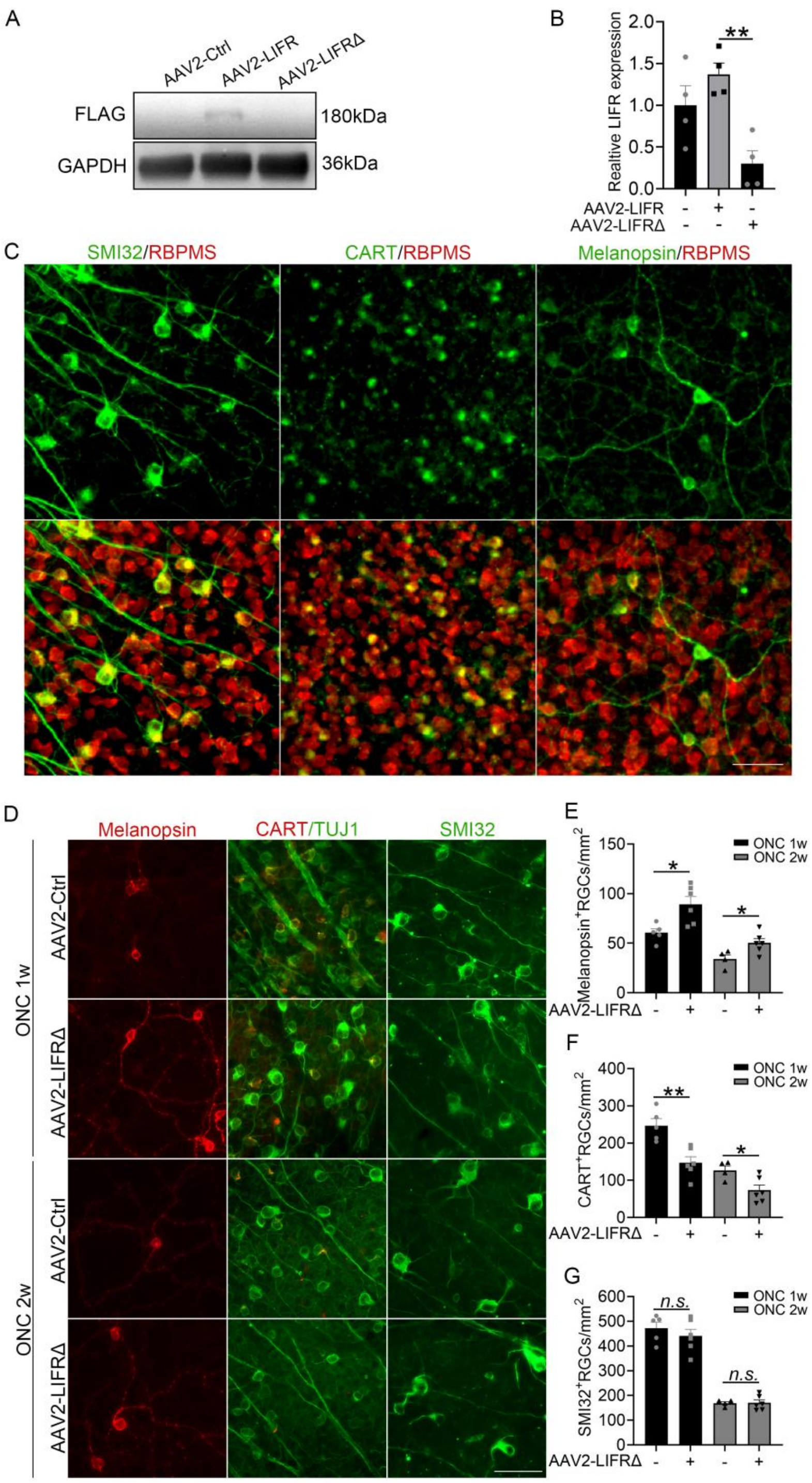
A
AAV2-Ctrl
AAV2-LIFR
AAV2-LIFRΔ
FLAG
180kDa
GAPDH
36kDa
B
Realtive LIFR expression
2.0
1.5
1.0
0.5
0.0
**
AAV2-LIFR - + -
AAV2-LIFRΔ - - +
C
SMI32/RBPMS
CART/RBPMS
Melanopsin/RBPMS
D
Melanopsin
CART/TUJ1
SMI32
ONC 1w
ONC 2w
AAV2-Ctrl
AAV2-LIFRΔ
AAV2-Ctrl
AAV2-LIFRΔ
E
Melanopsin+RGCs/mm2
ONC 1w
ONC 2w
150
100
50
0
*
*
AAV2-LIFRΔ - + - +
F
CART+RGCs/mm2
ONC 1w
ONC 2w
400
300
200
100
0
**
*
AAV2-LIFRΔ - + - +
G
SMI32+RGCs/mm2
ONC 1w
ONC 2w
600
500
400
300
200
100
0
n.s.
n.s.
AAV2-LIFRΔ - + - +

Supplemental Figure 9. LIFR intracellular domain alters RGC subtypes.

(A) Representative western blot showing FLAG-tagged full-length LIFR in retinal lysates two weeks after intravitreal injection of AAV2-Ctrl, AAV2-LIFR, or AAV2-LIFRΔ. GAPDH was used as the loading control. This experiment was conducted once.

(B) Quantification of relative FLAG/LIFR expression normalized to GAPDH. Statistical significance was determined by one-way ANOVA followed by Tukey's multiple-comparison test (** $P < 0.01$; n = 4 retinas per group).

(C) Whole-mounted retinas immunostained for SMI32 (*green*, left), CART (*green*, center), or Melanopsin (*green*, right), together with RBPMS (*red*). Scale bar, 50 µm. This experiment was conducted once.

(D) Whole-mounted retina stained for Melanopsin (*red*, left), CART (*red*, middle) or SMI32 (*green*, right) to visualize ipRGCs, ooDSGCs or αRGCs, respectively at 1 and 2 weeks post-nerve injury. Scale bar, 50 µm. This experiment was conducted once.

(E-G) Quantification of ipRGCs (E), CART-positive ooDSGCs (F), and SMI32-positive αRGCs (G) at 1 and 2 weeks post-ONC. Statistical significance was determined by unpaired two-tailed t test. (AAV2-LIFRΔ *vs.* AAV2-Ctrl, *n.s.*: $P > 0.05$, *$P < 0.05$, **$P < 0.01$; n=4-6 retinas per group).
Data represent mean ± SEM.
