# Supplementary material for: Optic nerve regeneration requires the intracellular domain of LIFRα/CD118
Source: JCI Insight. 2026 Sep 1;11(19):e202223. doi: 10.1172/jci.insight.202223 (PMC13726422; doi:10.1172/jci.insight.202223)
Supplement: Supplementary file 2 [file jciinsight-11-202223-s280.pdf]

Full unedited LIFR blot for Supplemental Figure 2C

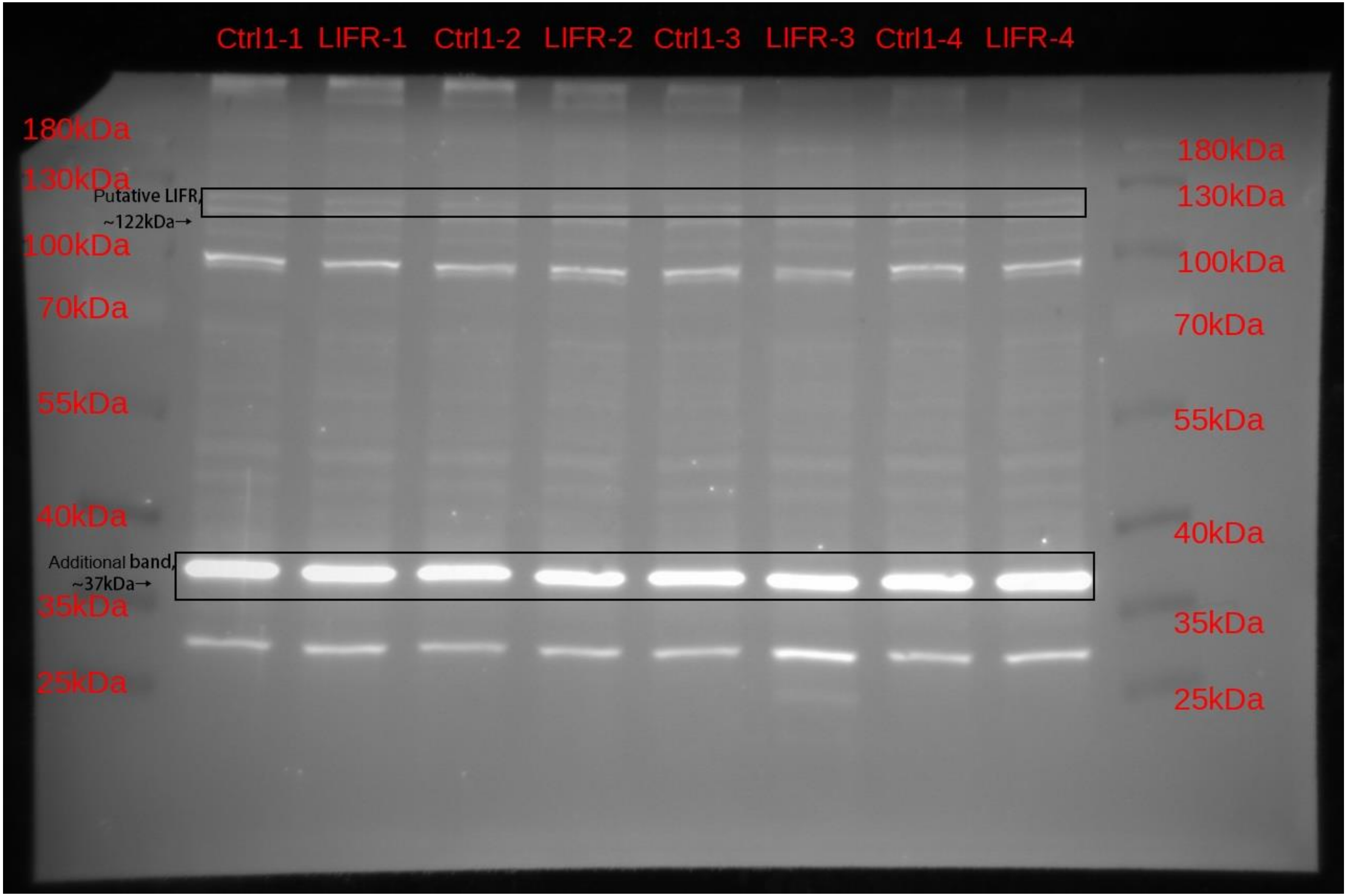

Lane 1, 3, 5, 7: AAV2-Ctrl; Lane 2, 4, 6, 8: AAV2-LIFR. Each lane represents an independent biological replicate. The upper boxed region indicates the putative LIFR band at ~122 kDa, and the lower boxed region indicates an additional band at ~37 kDa; both boxed regions are shown in Supplemental Figure 2C.

Full unedited Tublin blot for Supplemental Figure 2C

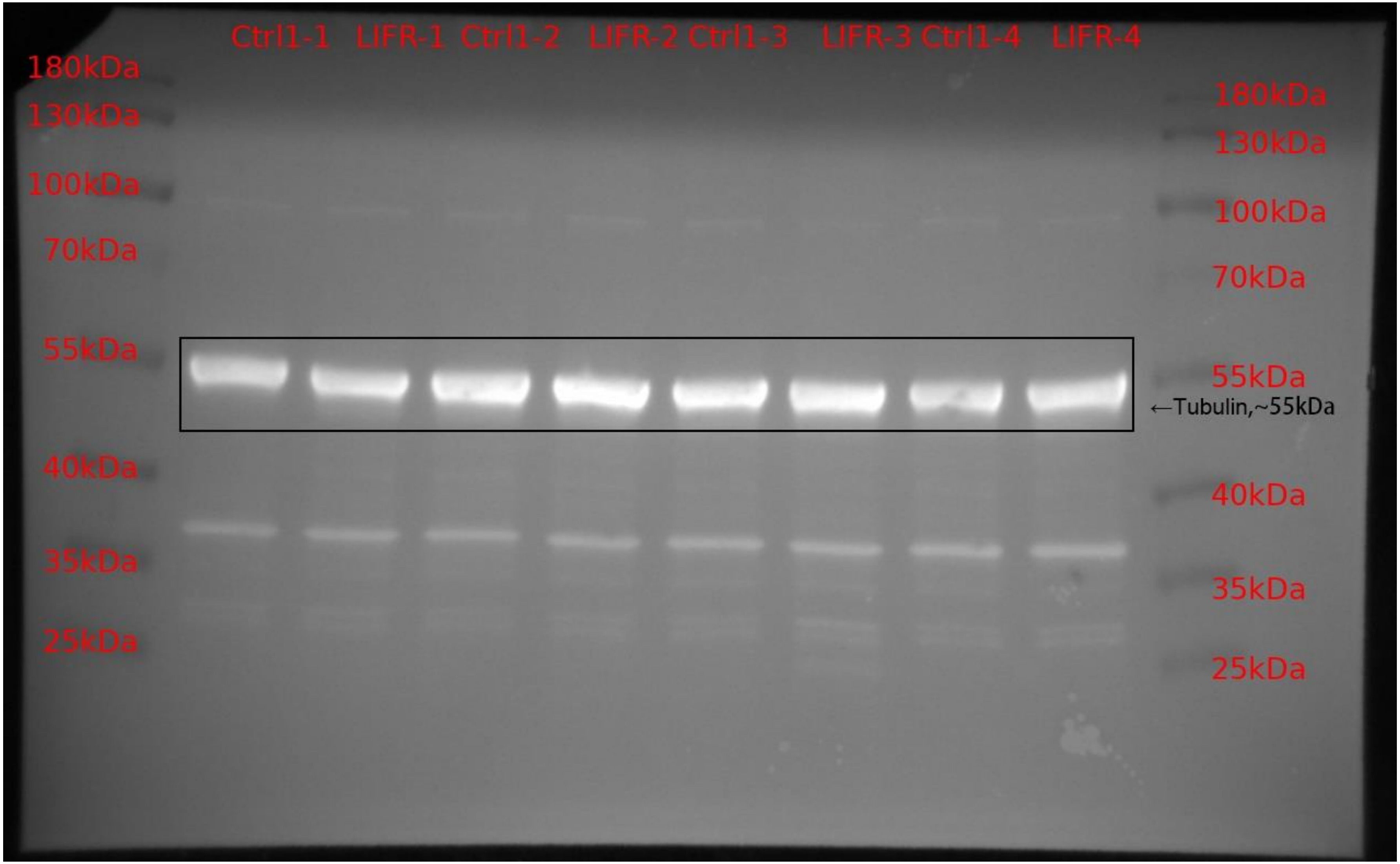

Lane 1, 3, 5, 7: AAV2-Ctrl; Lane 2, 4, 6, 8: AAV2-LIFR. Each lane represents an independent biological replicate. The boxed region indicates the Tubulin loading-control band at ~55 kDa shown in Supplemental Figure 2C.

Full unedited LIFR blot for Supplemental Figure 6A

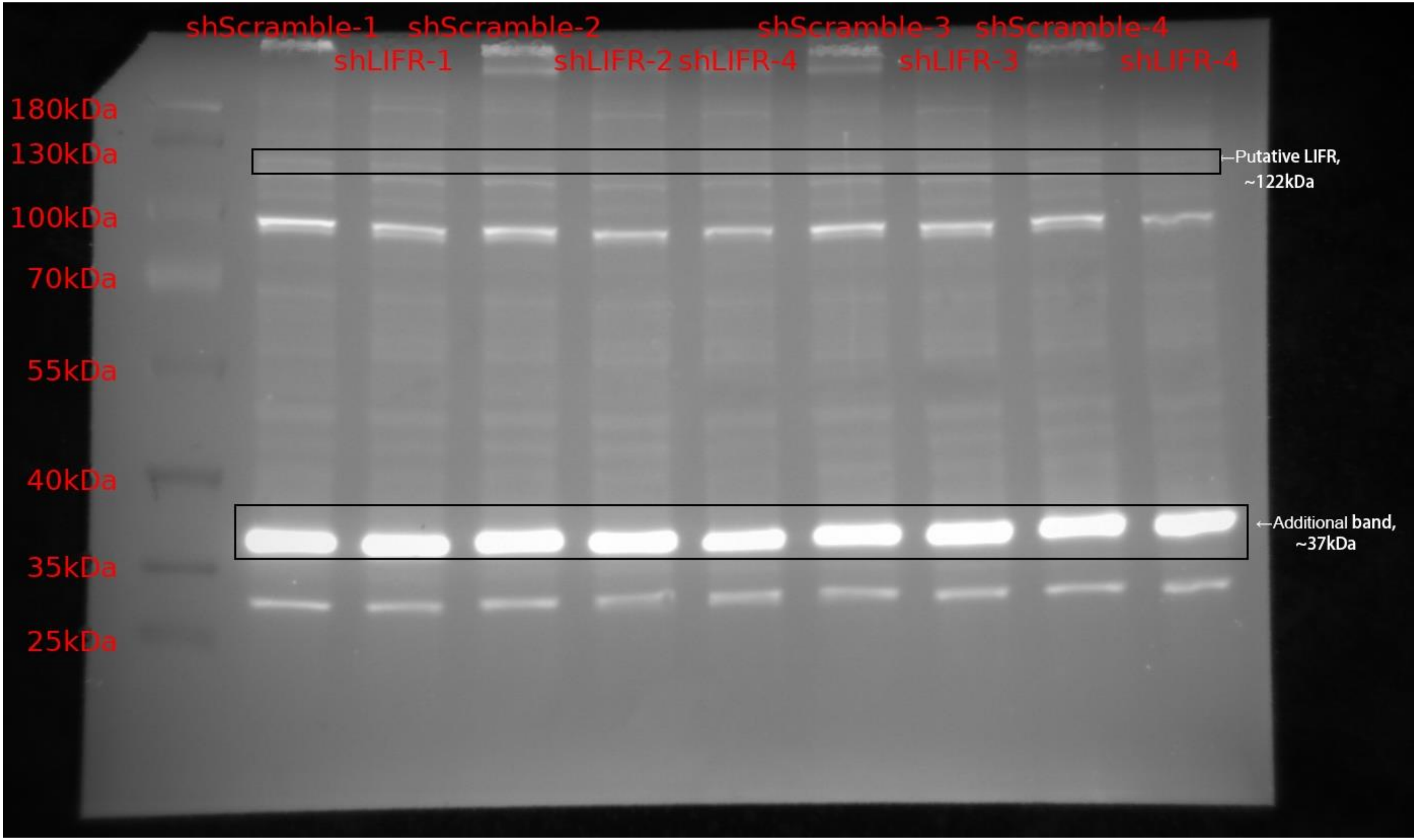

Lane 1, 3, 6, 8: AAV2-shScramble; Lane 2, 4, 5, 7, 9: AAV2-shLIFR. Lane 5 and Lane 9 are duplicate loading of the same AAV2-shLIFR-4 sample. The upper boxed region indicates the putative LIFR band at ~122 kDa, and the lower boxed region indicates an additional band at ~37 kDa; both boxed regions are shown in Supplemental Figure 6A.

Full unedited Tubulin blot for Supplemental Figure 6A

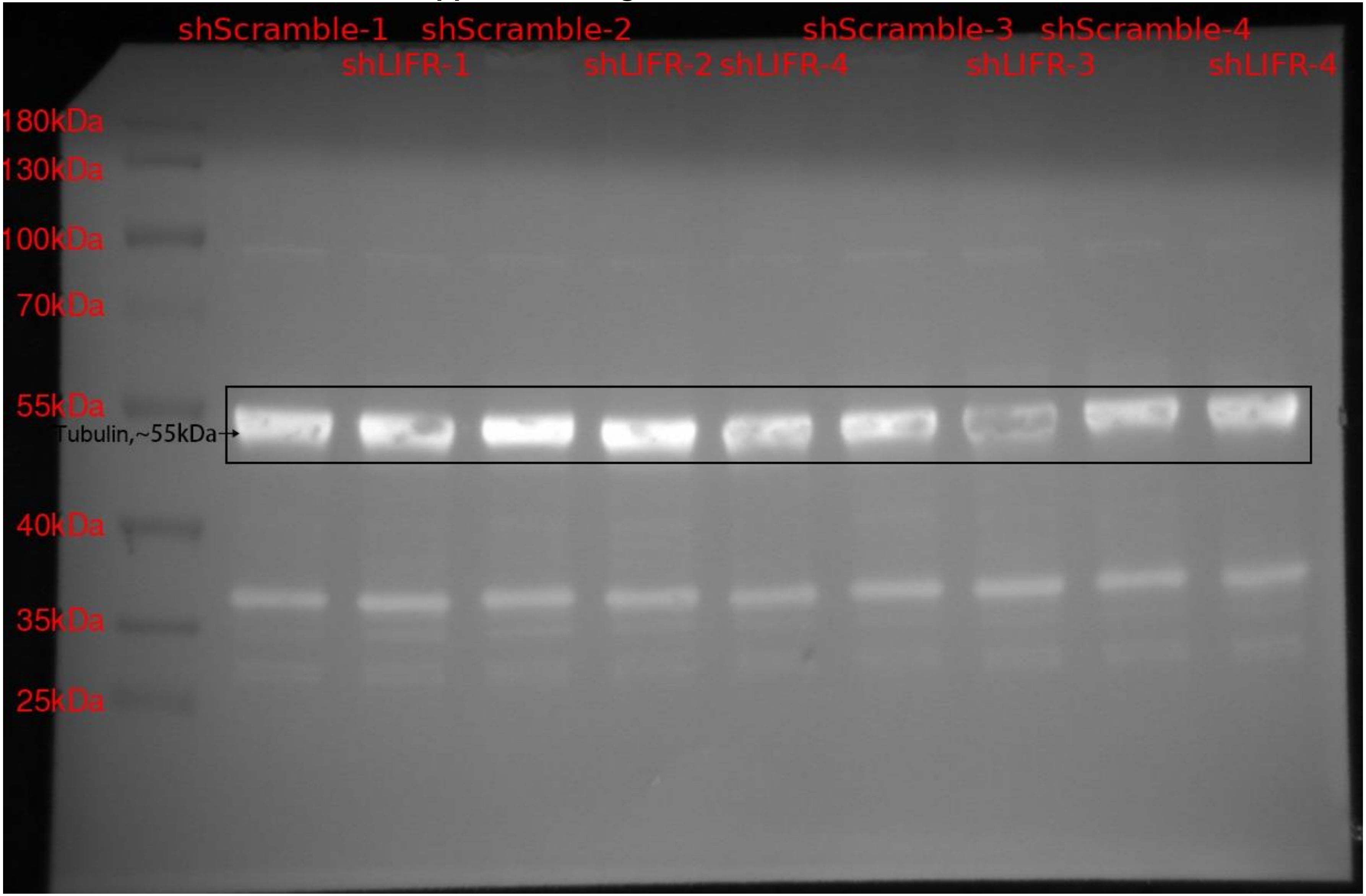

Lane 1, 3, 6, 8: AAV2-shScramble; Lane 2, 4, 5, 7, 9: AAV2-shLIFR. Lane 5 and Lane 9 represent duplicate loading of the same AAV2-shLIFR-4 sample; the other lanes represent independent biological replicates. The boxed region indicates the Tubulin loading-control band at ~55 kDa shown in Supplemental Figure 6A.

Full unedited FLAG blot for Supplemental Figure 8A

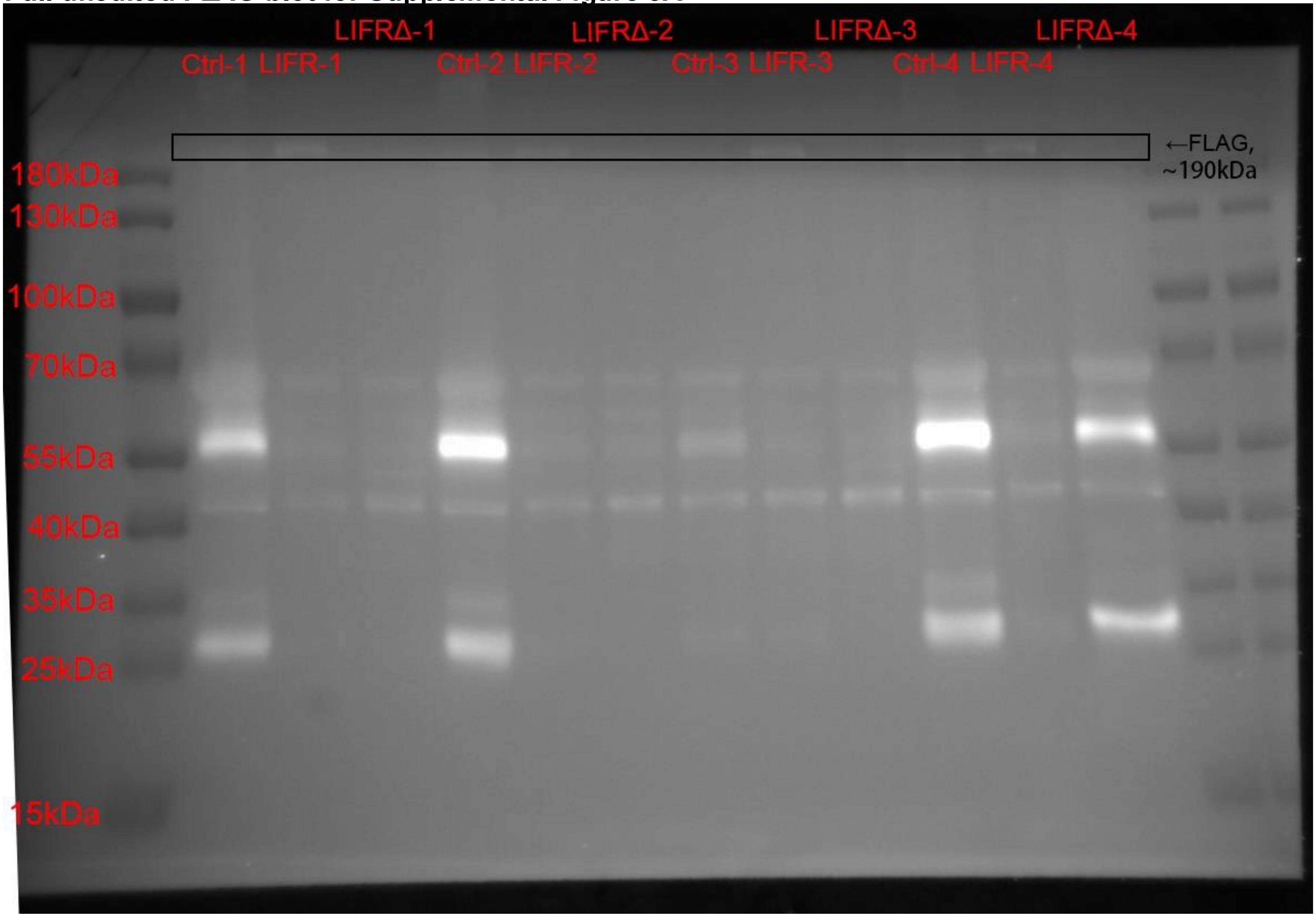

Lane 1, 4, 7, 10: AAV2-Ctrl; Lane 2, 5, 8, 11: AAV2-LIFR; Lane 3, 6, 9, 12: AAV2-LIFRΔ. Each lane represents an independent biological replicate. The boxed region indicates FLAG-tagged full-length LIFR at ~190 kDa shown in Supplemental Figure 8A.

Full unedited GAPDH blot for Supplemental Figure 8A

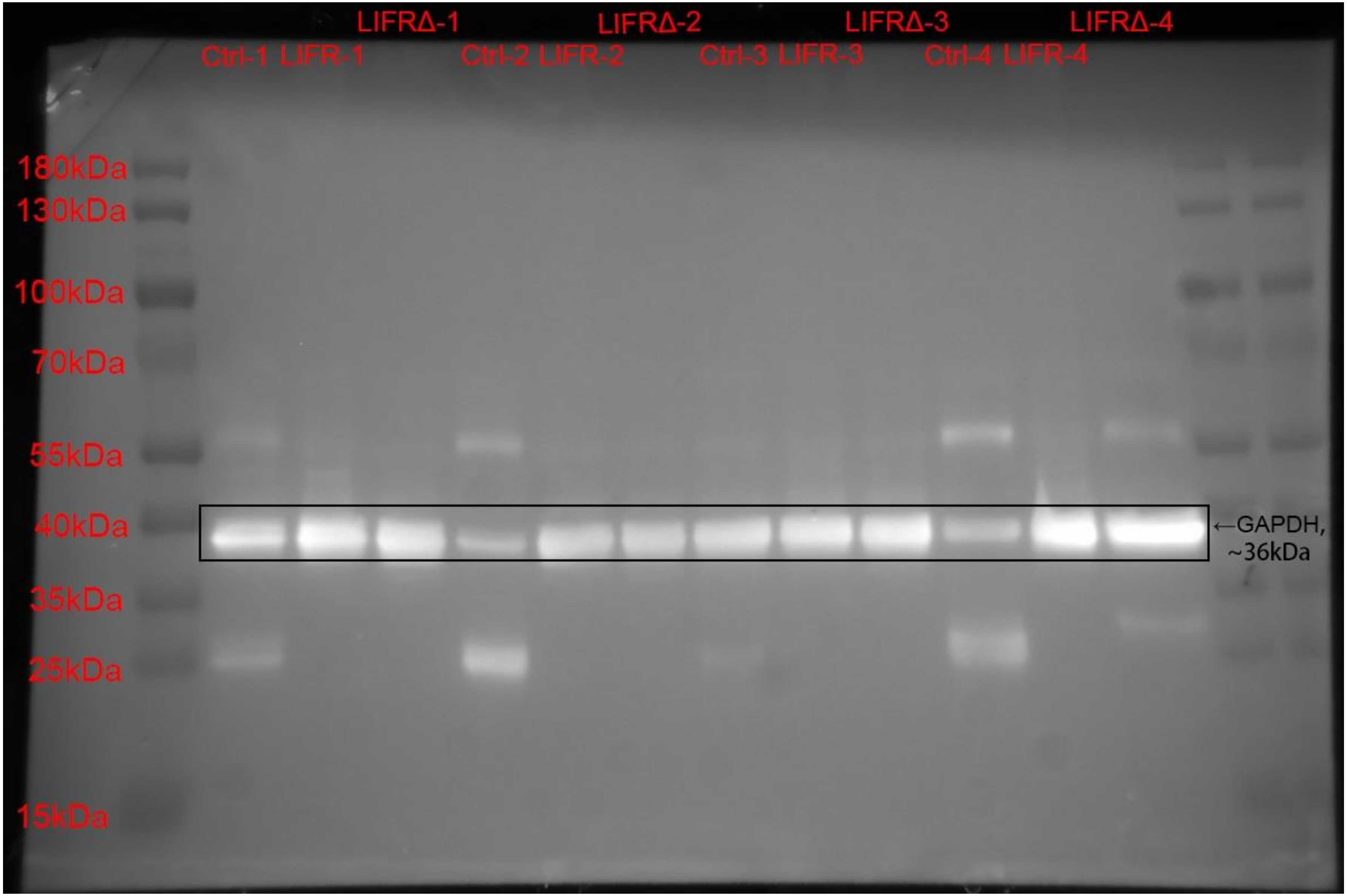

Lane 1, 4, 7, 10: AAV2-Ctrl; Lane 2, 5, 8, 11: AAV2-LIFR; Lane 3, 6, 9, 12: AAV2-ΔLIFR. Each lane represents an independent biological replicate. The boxed region indicates the GAPDH loading-control band at ~36 kDa shown in Supplemental Figure 8A.
